# Non-Majorana origin of anomalous current-phase relation and Josephson diode effect in Bi_2_Se_3_/NbSe_2_ Josephson junctions

**DOI:** 10.1126/sciadv.adw6925

**Published:** 2025-06-13

**Authors:** Andrei Kudriashov, Xiangyu Zhou, Razmik A. Hovhannisyan, Alexander S. Frolov, Leonid Elesin, Yi Bo Wang, Ekaterina V. Zharkova, Takashi Taniguchi, Kenji Watanabe, Zheng Liu, Kostya S. Novoselov, Lada V. Yashina, Xin Zhou, Denis A. Bandurin

**Affiliations:** ^1^ Department of Materials Science and Engineering, National University of Singapore, Singapore, Singapore.; ^2^ Institute for Functional Intelligent Materials, National University of Singapore, Singapore, Singapore.; ^3^ Department of Physics, Stockholm University, AlbaNova University Center, SE-10691 Stockholm, Sweden.; ^4^ Chemistry Department, M.V. Lomonosov Moscow State University, Moscow, Russia.; ^5^ Moscow Center for Advanced Studies, Moscow, Russia.; ^6^ Programmable Functional Materials Lab, Center for Neurophysics and Neuromorphic Technologies, Moscow 127495, Russia.; ^7^ International Center for Materials Nanoarchitectonics, National Institute of Material Science, Tsukuba 305-0044, Japan.; ^8^ Research Center for Functional Materials, National Institute of Material Science, Tsukuba, Japan.; ^9^ School of Materials Science and Engineering, Nanyang Technological University, Singapore, Singapore.

## Abstract

Josephson junctions (JJs) are key to superconducting quantum technologies and the search for self-conjugate quasiparticles potentially useful for fault-tolerant quantum computing. In topological insulator (TI)–based JJs, measuring the current-phase relation (CPR) can reveal unconventional effects such as Majorana bound states (MBS) and nonreciprocal transport. However, reconstructing CPR as a function of magnetic field has not been attempted. Here, we present a platform for field-dependent CPR measurements in planar JJs made of NbSe_2_ and few-layer Bi_2_Se_3_. When a flux quantum $\Phi_{0}$ threads the junction, we observe anomalous peak-dip CPR structure and nonreciprocal supercurrent flow. We show that these arise from a nonuniform supercurrent distribution that also leads to a robust and tunable Josephson diode effect. Furthermore, despite numerous previous studies, we find no evidence of MBS. Our results establish magnetic field–dependent CPR as a powerful probe of TI-based superconducting devices and offer design strategies for nonreciprocal superconducting electronics.

## Teaser

Current-phase relation of Bi_2_Se_3_ Josephson junctions reveals no Majorana-bound states but shows non-reciprocal supercurrent.

## INTRODUCTION

Characterizing and controlling supercurrent flow in Josephson junctions (JJs) is critical for advancing both fundamental research and practical applications, from superconducting classical and quantum technologies (*1*–*4*) to the discovery of exotic quasiparticles (*5*–*11*). While conventional methods, such as measuring the critical current between dissipationless and resistive states, have been instrumental in studying JJs, they often provide limited insight into fundamental mechanisms like spin-orbit coupling (SOC) (*12*), quantum-geometric effects (*13*), and pairing symmetry (*14*) that govern their superconducting properties. At the same time, spectroscopic techniques probing the amplitude of the superconducting wave function (*15*) lack direct access to phase-dependent phenomena such as current-induced hidden states (*16*) and screening currents in superconductor/ferromagnet hybrids (*17*) that were uncovered only recently with the advent of sensitive noninvasive scanning probes. These limitations are amplified in JJs with topologically nontrivial weak links (*18*–*21*), where multiple confounding factors (*22*–*30*) can mimic transport signatures of Majorana bound states (MBSs) (*31*–*34*), pivotal for fault-tolerant quantum computing (*5*, *35*, *36*). Phase-sensitive information is also crucial for understanding nonreciprocal supercurrent transport and the Josephson diode effect (JDE), a research focus in superconducting nanoelectronics (*37*). While JDE has been observed in various systems (*17*, *37*–*41*), directly probing the direction and amplitude of nonreciprocal supercurrent in topological insulator (TI)–based JJs as a function of external tuning knobs (*42*)—such as a magnetic field—and uncovering its possible relation to MBS (*43*, *44*) have remained experimentally difficult. These challenges have resulted in a proliferation of studies making unsubstantiated claims about MBS signatures in some TIs and the nature of the JDE in related systems. To circumvent the limitations of the transport and spectroscopic approaches and get access to the internals of the topological JJs, an alternative methodology framework is needed.

Here, we propose and realize such a framework that is based on accurate field-dependent reconstruction of the current-phase relation (CPR) in a lateral JJ made of a superconducting NbSe_2_ and one of the most-known TI—Bi_2_Se_3_ (*45*, *46*). Although several studies have investigated the zero-field CPR of TI-based JJs (*47*–*52*), none of them reported the exploration of the critical regime near $\Phi_{0}$ , where MBSs are anticipated to dominate the supercurrent (*32*, *33*). At the same time, the CPR of conventional and topological JJs in the regime of JDE has remained largely unexplored. We fabricated lateral van der Waals (vdW) heterojunctions and incorporated them into superconducting quantum interference devices (SQUIDs). By controlling the flux through the SQUID using a superconducting local flux line, we performed precise CPR measurements of individual JJs and its dependence on the external magnetic field. Near $\Phi_{0}$ , we observe unconventional CPR characteristics and nonreciprocal supercurrent flow, which we show to stem from the nonuniform supercurrent distribution (SD). The latter generates distinctive nonreciprocal CPR, giving rise to a robust and tunable JDE with 30% efficiency. Finally, we observe no signatures of MBS near a single flux quantum conditions in the CPR data, thereby challenging dozens of theoretical and experimental studies on superconductor-proximitized Bi_2_Se_3_.

## RESULTS

### Planar vdW JJs

**Fig. 1.**
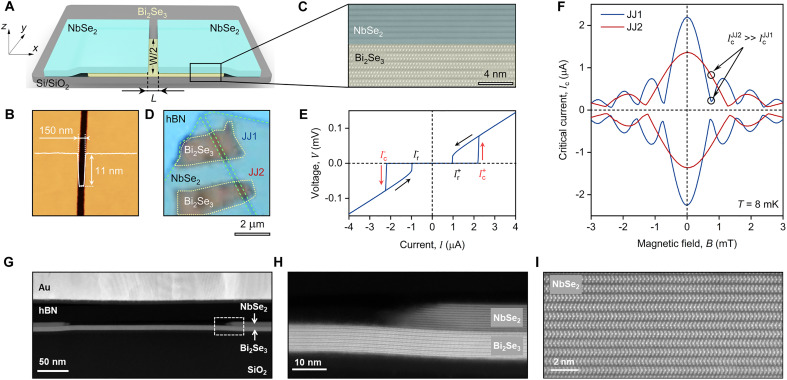
Planar vdW NbSe_2_/Bi_2_Se_3_ JJs. (**A**) Schematic illustration of a single JJ. A few-layer Bi_2_Se_3_ film is covered by a cracked NbSe_2_ flake, with Ti/Au electrodes contacting the NbSe_2_ regions. The device is fabricated on an oxidized Si substrate. (**B**) Atomic force microscopy topography of the cracked NbSe_2_ region. White solid line indicates the height profile across the crack. Measurements performed in an Ar-filled glovebox. (**C**) Representative high-resolution HAADF-STEM image across the NbSe_2_/Bi_2_Se_3_ interface. (**D**) Optical micrograph of an hBN-encapsulated NbSe_2_/Bi_2_Se_3_ stack comprising two Bi_2_Se_3_ flakes that form two JJs (JJ1 and JJ2). (**E**) Characteristic I-V curve of JJ1 measured at base temperature T=8 mK. Arrows indicate the bias current sweep direction. $I_{c}^{+,-}$ and $I_{r}^{+,-}$ denote critical and retrapping currents for positive (+) and negative (−) current directions, respectively. (**F**) Magnetic field dependence of the critical current $I_{c}$ for JJ1 (blue) and JJ2 (red) at the specified temperature. (**G**) Typical STEM image of the device cross section. (**H**) Zoomed-in STEM image of the NbSe_2_/Bi_2_Se_3_ interface at the end of the NbSe_2_ flake. (**I**) STEM image of the atomic planes in NbSe_2_.

Our devices consist of planar JJs formed by two superconducting 2H-NbSe_2_ electrodes coupled through a 5-nm-thick TI Bi_2_Se_3_ (Fig. 1A) (*53*). Device fabrication was performed using a standard dry transfer method within an argon-filled glovebox to prevent surface degradation of the constituent materials (*54*). The fabrication process began with mechanical exfoliation of thin Bi_2_Se_3_ flakes onto a Si/SiO_2_ substrate. Subsequently, an atomically flat NbSe_2_ flake, containing an intrinsic crack (*55*, *56*), was transferred onto the Bi_2_Se_3_, forming two superconducting electrodes separated by a narrow gap of L≈150 nm (Fig. 1B). Unlike conventional thin-film deposition techniques that typically involve sputtering superconducting electrodes onto TIs—a process known to introduce structural and compositional disorder (*57*)—our method ensures atomically sharp interface between the materials critical for superconducting proximity (*58*–*60*). High-resolution high-angle annular dark-field scanning transmission electron microscopy (HAADF-STEM) imaging (Fig. 1, C and G to I, and Supplementary Materials) reveals the structural characteristics of the heterostructure, confirming the exceptional interface quality, which is important for JJ experiments. The heterostructure was further covered by a relatively thick (40 nm) slab of hexagonal boron nitride (hBN) to protect the device during subsequent patterning using electron beam lithography (see Materials and Methods and the Supplementary Materials).

We fabricated three different samples, all featuring robust proximity effect (Supplementary Materials). The data in the main text are shown for one of them. This device contains two junctions, which we will refer to as JJ1 and JJ2, shown in the optical image in Fig. 1D. We intentionally oriented NbSe_2_ crack in a way to ensure different junction widths and patterned the device in a SQUID geometry, which is required for CPR measurements (details below). While we first measured the as-fabricated SQUID, for convenience, we initially present data from the individual junctions, obtained after etching the SQUID into two independent devices. The current-voltage ( I-V ) curve of JJ1, shown in Fig. 1E, is typical for proximity JJs. It displays a zero-voltage state for currents below the critical current $I_{c}$ , followed by a sharp transition to the resistive state as the current exceeds $I_{c}$ . Upon decreasing the current, the junction remains in the resistive state until the current drops below the retrapping current $I_{r}$ , leading to hysteresis in the curve. Hysteresis is typical for this type of device and is likely caused by a combination of self-heating effects (*61*) and an increased McCumber parameter, which can become large due to the enhanced capacitance from the gate electrodes (*62*).

Since the transition from superconducting to normal state is very sharp, it allows us to define a critical current $I_{c}$ and a retrapping current $I_{r}$ using the threshold method and accurately determine its dependence on the magnetic field, B. Figure 1F shows $I_{c}$ as a function of B for both JJ1 and JJ2. It exhibits a characteristic Fraunhofer-like pattern, where the critical current $I_{c}$ oscillates as a function of the applied magnetic field B , reaching minimum (nonzero) values when the magnetic flux through the junctions is close to integer multiples of the magnetic flux quantum, $\Phi_{1,2}=n\Phi_{0}$ , where 1,2 indices correspond to JJ1 and JJ2, respectively, and n is integer.

### CPR measurements

Since the widths of the two junctions were intentionally made different, their $I_{c}\left(B\right)$ patterns also differ (as shown in Fig. 1, D and F). Importantly, when B≈0.75 mT (which corresponds to the interference pattern minimum), $I_{c}^{\text{JJ}1}\approx 60$ nA and $I_{c}^{\text{JJ}2}\approx 1\;$ μA. By redesigning the asymmetric SQUID technique (*51*, *63*, *64*) with an additional flux bias, we leveraged this order-of-magnitude asymmetry in critical current to enable measurements of the CPR of JJ1 as a function of external magnetic field, a capability previously unattained in TI-based JJs.

The idea behind this technique is the following. The supercurrent through the SQUID is the sum of supercurrents through individual JJs: $I_{s}^{\text{SQUID}}=I_{s}^{\text{JJ}1}\left(\varphi_{1}\right)+I_{s}^{\text{JJ}2}\left(\varphi_{2}\right)$ , where $\varphi_{1}$ and $\varphi_{2}$ are the phase differences in JJ1 and JJ2, respectively. Assuming negligible inductance L of the SQUID (in our geometry $L\cdot I_{c}/\Phi_{0}∼10^{-3}$ ), the phase differences are related by the magnetic flux through the SQUID, Φ , as $\varphi_{1}-\varphi_{2}=2\pi \Phi /\Phi_{0}$.

When the critical current of JJ2 is notably larger than that of JJ1, $\varphi_{2}=\varphi^{*}$ , where $\varphi^{*}$ is the phase at which the critical current of JJ2 is achieved and it is almost independent of Φ . As a result, the critical current of the SQUID can be expressed as

$$I_{c}^{\text{SQUID}}\left(\Phi \right)=I_{c}^{\text{JJ}2}+I_{s}^{\text{JJ}1}\left[\varphi_{1}\left(\Phi \right)\right]$$ (1)

where $\varphi_{1}\left(\Phi \right)=\varphi^{*}+2\pi \Phi /\Phi_{0}$ . Therefore, the desired CPR, $I_{s}^{\text{JJ}1}\left(\varphi_{1}\right)$ , can be reconstructed from Eq. 1 through the accurate measurements of $I_{c}^{\text{SQUID}}\left(\Phi \right)$ (*51*).

**Fig. 2.**
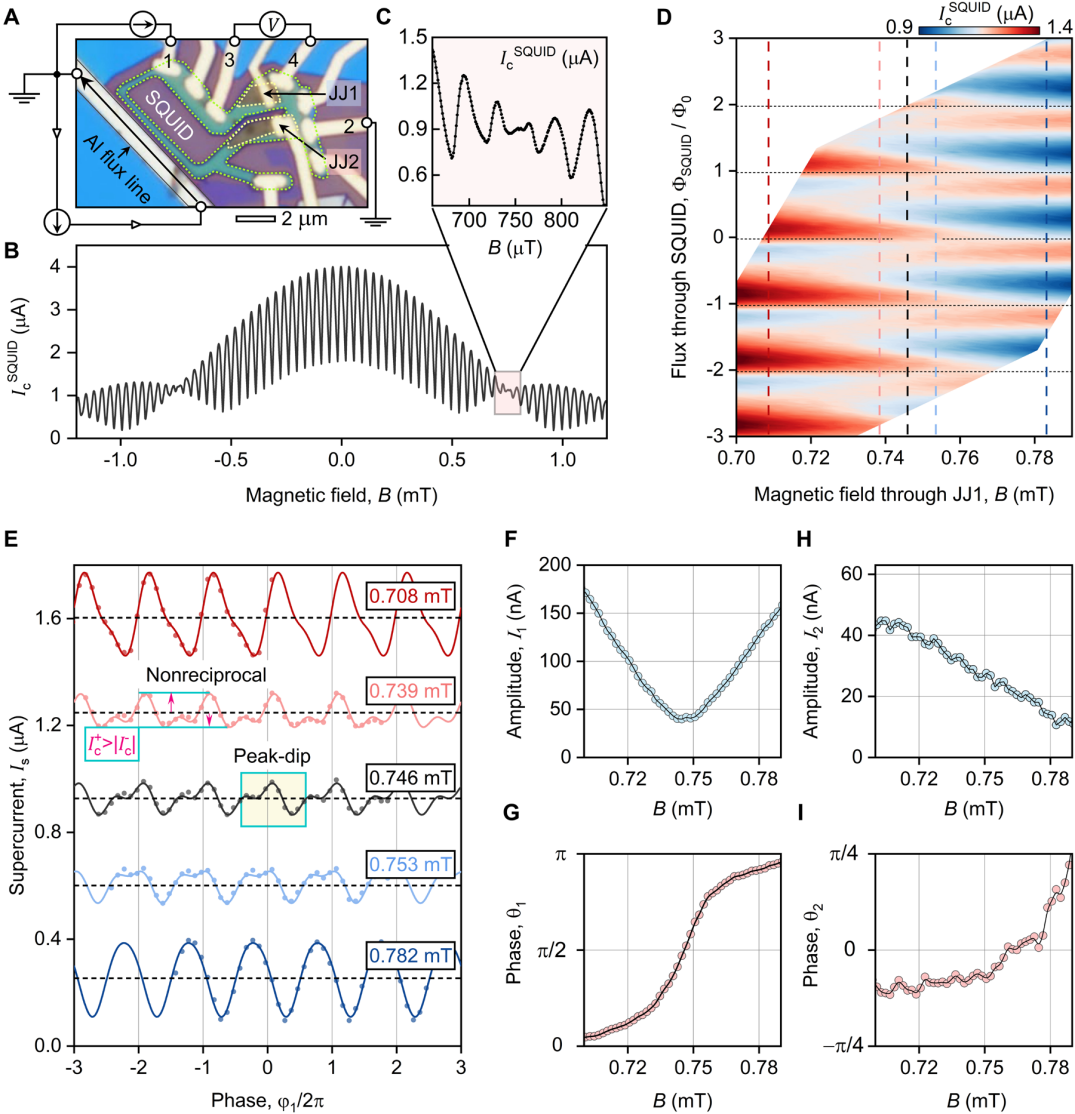
NbSe_2_/Bi_2_Se_3_ SQUID and CPR measurements. (**A**) Optical micrograph of the SQUID incorporating two JJs (JJ1 and JJ2) with measurement configuration overlay. Direct current flows between contacts 1 and 2, while voltage is measured across contacts 3 and 4. A perpendicular magnetic field is applied externally, and a superconducting Al line controls the magnetic flux through the SQUID. (**B**) Critical current $I_{c}^{\text{SQUID}}$ as a function of magnetic field, measured at base temperature T=8 mK. (**C**) Magnified view of the region near one magnetic flux quantum through JJ1. (**D**) $I_{c}^{\text{SQUID}}$ plotted against magnetic field ( B ) and magnetic flux through SQUID ( Φ ), normalized to $\Phi_{0}$ . (**E**) CPR of JJ1 extracted along vertical dashed lines in (D). Symbols represent experimental data; solid lines show best fits to $I_{s}^{\text{JJ}1}\left(\varphi_{1}\right)=I_{0}+I_{1}\text{sin}\left(\varphi_{1}+\theta_{1}\right)+I_{2}\text{sin}\left(2\varphi_{1}+\theta_{2}\right)$ . Dashed lines represent $I_{0}$ . Data are shifted vertically for clarity. (**F**) Amplitude $I_{1}$ of the first harmonic plotted versus magnetic field B . (**G**) Phase $\theta_{1}$ of the first harmonic plotted versus magnetic field B . (**H**) Amplitude $I_{2}$ of the second harmonic plotted versus magnetic field B . (**I**) Phase $\theta_{2}$ of the second harmonic plotted versus magnetic field B.

To perform such experiment, we used a SQUID made of JJ1 and JJ2 shown in Fig. 2A. Figure 2B reveals fast $I_{c}^{\text{SQUID}}\left(B\right)$ oscillations modulated by the combination of interference patterns from individual JJs, highlighting the typical SQUID pattern. When B≈0.746 mT, the amplitude of the oscillations is suppressed (Fig. 2C), reflecting that JJ1 is close to the Fraunhofer minimum—the point of interest in our study. For an accurate control of Φ while maintaining magnetic field through JJ1 constant, we endowed our device with an aluminum flux line located relatively far from the JJs (Fig. 2A). By applying the current through the flux line and accounting for the superposition of both magnetic fields, we mapped the full dependence of $I_{c}^{\text{SQUID}}$ on Φ and B shown in Fig. 2D.

Next, using these data and Eq. 1, we obtained the CPR $I_{s}^{\text{JJ}1}\left(\varphi_{1}\right)$ at selected values of B in the vicinity of the first interference pattern minimum, as shown in Fig. 2E. This is the central plot of our study. At B=0.708 mT and B=0.782 mT, the CPRs are 2π-periodic functions oscillating in anti-phase with respect to each other. This phase shift is expected since the JJs experience 0-π transition when crossing the integer number of flux quanta (*25*). At B=0.746 mT, the periodic pattern undergoes a notable transformation, developing an anomalous peak-dip structure (highlighted by the yellow rectangle) that notably deviates from the anticipated CPR behavior where the critical current through JJ1 is expected to be fully suppressed (see below).

For further analysis of the observed anomalies, we characterize these features phenomenologically and fit the data with a two-harmonic expansion: $I_{s}^{\text{JJ}1}\left(\varphi_{1}\right)=I_{0}+I_{1}\text{sin}\left(\varphi_{1}+\theta_{1}\right)+I_{2}\text{sin}\left(2\varphi_{1}+\theta_{2}\right)$ (solid lines in Fig. 2E). Here, $I_{1,2}$ and $\theta_{1,2}$ represent the amplitudes and phases of the first and second harmonics, respectively, and $I_{0}$ is the offset introduced by the asymmetric SQUID technique (see Eq. 1). The amplitude of the first harmonic $I_{1}$ decreases, reaching a minimum nonzero value at B=0.746 mT, before increasing again, while the phase $\theta_{1}$ undergoes a smooth 0-π transition, as shown in Fig. 2 (F and G). In contrast, the amplitude of the second harmonic $I_{2}$ gradually decreases over the entire measurement interval, showing no features associated with B=0.746 mT, while the phase $\theta_{2}$ remains close to zero, as shown in Fig. 2 (H and I). Therefore, there is a magnetic field–dependent phase shift between first and second harmonics, which leads to another notable characteristic of the observed CPR: its directional asymmetry (the amplitudes of positive and negative supercurrents differ at certain magnetic fields, as marked in Fig. 2E). This asymmetry implies that the junction exhibits a nonreciprocal transport, where the magnitude of the critical current depends on its direction through the JJ.

### Nonreciprocal CPR and Josephson diode

**Fig. 3.**
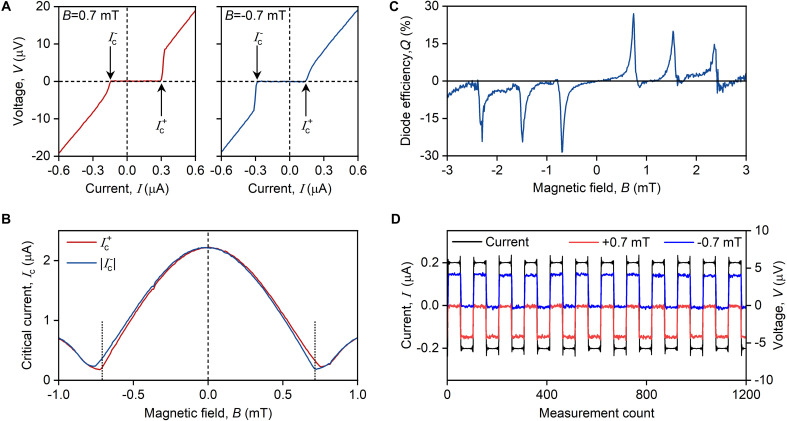
Josephson diode in TI-based JJs. (**A**) I-V curves of JJ1 measured at specified magnetic fields B . (**B**) Positive ( $I_{c}^{+}$ ) and negative ( $I_{c}^{-}$ ) critical currents for JJ1 as a function of B . Vertical dotted lines correspond to B=±0.7 mT. (**C**) Superconducting diode efficiency, Q , as a function of B . (**D**) Rectified voltage across JJ1 (right axis) measured by its excitation with the square-wave current (left axis) with the amplitude 0.2 μA for B=0.7 mT (red curve) and B=−0.7 mT (blue curve). T=8 mK.

To demonstrate and analyze the nonreciprocal behavior, we return to the data obtained on JJ1. Figure 3A shows examples of two I-V curves for JJ1 measured at B=±0.7 mT and reveals the anticipated nonreciprocity: $I_{c}^{+}\neq I_{c}^{-}$ , indicating the manifestation of JDE (*37*). Figure 3B details this observation further by showing $I_{c}^{+}$ and $I_{c}^{-}$ as a function of B in the vicinity of the single flux quantum. The clear in-equivalence between the two persists for ∣B∣<0.8 mT and abruptly disappears for larger ∣B∣.

For further analysis, we quantify the strength of the diode effect by introducing a diode efficiency factor $Q=\left(I_{c}^{+}-|\;I_{c}^{-}\;|\right)/\left(I_{c}^{+}+|\;I_{c}^{-}\;|\right)$. Figure 3C shows the tunability of Q on magnetic field B , revealing a distinctive tooth-like structure. The efficiency ∣Q∣ exhibits periodic behavior, vanishing at integer numbers of flux quanta $\Phi_{0}$ through the junction, followed by sharp increases with increasing B . In our devices, the maximum achieved efficiency ∣Q∣ reached approximately 30%.

These large values of ∣Q∣ allow us to demonstrate a robust rectification effect (Fig. 3D). To this end, we applied square-wave current oscillations (black line) across the junction with the amplitude 0.2 μA, which is between $|\;I_{c}^{-}\;|=0.18$ μA and $I_{c}^{+}=0.3$ μA. The resulting voltage drop appears only during half of the excitation period, and its sign can be controlled by the direction of B (see blue and red curves in Fig. 3D).

### Theoretical modeling

To understand the origin of the observed anomalous CPR and the JJ diode effect, we use the standard expression (*26*, *30*, *65*) that describes the supercurrent flow through the narrow JJ, i.e., when $W<\lambda_{J}$ , where W is the width of the junction and $\lambda_{J}$ is the Josephson penetration length (see the Supplementary Materials)

$$I_{s}\left(\varphi_{1}\right)=\int_{-W/2}^{W/2}J_{c}\left(y\right)j_{s}\left(\alpha \mathrm{By}+\varphi_{1}\right)dy$$ (2)

Here, y is the coordinate along the junction, with y=0 being the center of the junction, $J_{c}\left(y\right)$ is the SD at B=0 , $j_{s}$ is the local CPR, $\varphi_{1}$ is the Josephson free phase, $\alpha =2\pi L_{\text{eff}}/\Phi_{0}$ , and $L_{\text{eff}}$ is the effective length of the junction, which is determined by the geometry of the superconducting leads in the case of a planar JJ (*66*). Since $I_{s}\left(\varphi_{1}\right)$ describes the total supercurrent through the junction as a function of the phase difference between the superconducting leads $\varphi_{1}$ , we will refer to this dependence as a global CPR, the desired property of the Bi_2_Se_3_/NbSe_2_ JJ that we investigate in our study. Equation 2 allows to calculate the magnetic field dependence of the CPR as well as of the critical currents $I_{c}^{+}=\text{max}I_{s}\left(\varphi_{1}\right)$ and $I_{c}^{-}=\text{min}I_{s}\left(\varphi_{1}\right)$.

**Fig. 4.**
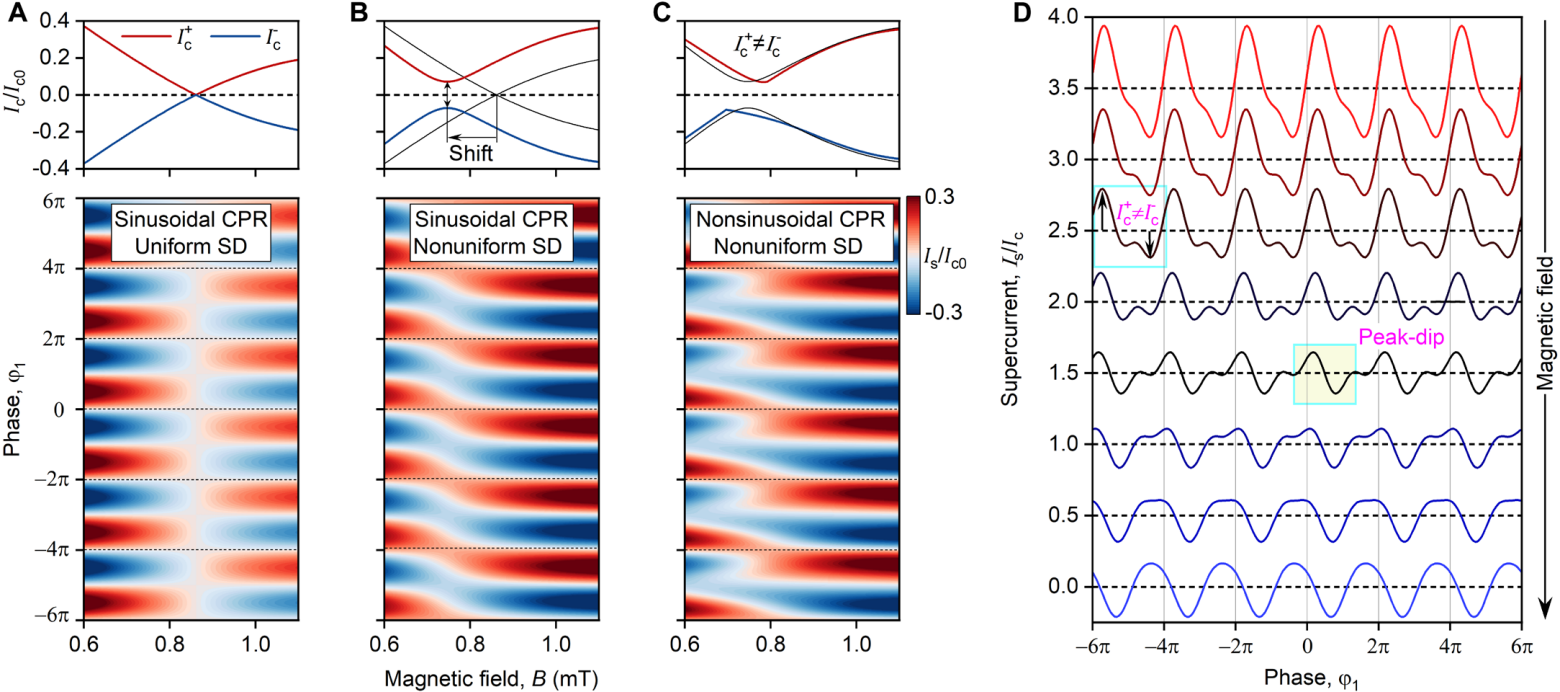
Theoretical modeling of the CPR. (**A** to **C**) Calculated positive $I_{c}^{+}$ and negative $I_{c}^{-}$ critical currents of the JJ as a function of magnetic field B (top) and calculated supercurrent $I_{s}$ as a function of phase difference $\varphi_{1}$ and magnetic field B (bottom). (A) Case of sinusoidal CPR and uniform SD. (B) Case of sinusoidal CPR and nonuniform SD. Black line in the top is the same as red and blue curves in (A). (C) Case of nonsinusoidal CPR and nonuniform SD. Black line in the top is the same as red and blue curves in (B). (**D**) Calculated $I_{s}\left(\varphi_{1}\right)$ for different magnetic fields B around Fraunhofer minima for the case shown in (C). The case of nonsinusoidal $j_{s}\left(\varphi_{1}\right)$ combined with a uniform current distribution is shown in the Supplementary Materials.

First, we apply Eq. 2 to the simplest case of uniform supercurrent flow $J_{c}\left(y\right)=1$ and sinusoidal local CPR $j_{s}\left(\varphi \right)=\text{sin}\left(\varphi \right)$ . The results are shown in Fig. 4A (bottom) that maps $I_{s}$ against B and $\varphi_{1}$ . The map reveals a sudden change of $I_{s}$ sign at $\Phi_{1}=\Phi_{0}$ that is achieved when B=0.85 mT, demonstrating the standard 0-π transition. The calculated dependence is drastically different from the experimentally obtained CPR (see Fig. 2D), highlighting the distinctive supercurrent transport mechanism in our devices.

**Fig. 5.**
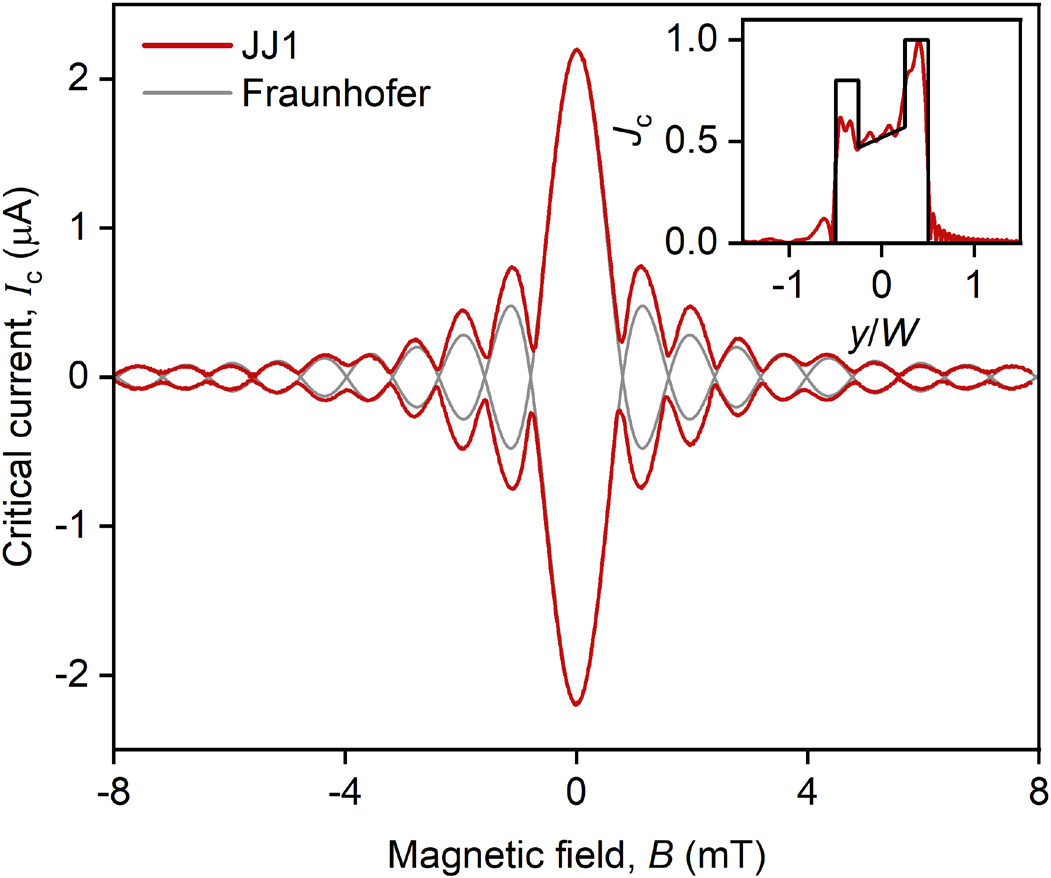
Reconstruction of the SD. Critical current $I_{c}$ of JJ1 as a function of magnetic field B . The inset shows the SD, which was reconstructed from $I_{c}\left(B\right)$ , by the red line, and the SD, which was used for modeling, by the black line.

The obtained $I_{c}^{+,-}\left(B\right)$ dependencies, shown in Fig. 4A (top), describe conventional Fraunhofer pattern $I_{c}=I_{c0}|\;\text{sin}\left(\pi \Phi /\Phi_{0}\right)/\left(\pi \Phi /\Phi_{0}\right)\;|$ for ideal JJs, which notably deviate from experimentally measured $I_{c}\left(B\right)$ (Fig. 5). This implies that our device features a nonuniform SD $J_{c}\left(y\right)$ along the junction that needs to be accounted for in further analysis. To this end, we determine the SD $J_{c}\left(y\right)$ using a standard method that calculates the Fourier transform of the measured $I_{c}\left(B\right)$ dependencies (*22*). The results are shown by the red line in the inset of Fig. 5 that reveals (i) increased $J_{c}$ at the end of the JJ and (ii) the linear slope of $J_{c}\left(y\right)$ across the whole junction.

To take into account the nonuniform SD, for simplicity, we approximate the extracted $J_{c}\left(y\right)$ with a model dependence (black curve in Fig. 5) and use it in Eq. 2. The resulting $I_{c}\left(B\right)$ and $I_{s}\left(B,\varphi_{1}\right)$ dependencies are shown in Fig. 4B. Compared with the previous case, nonuniform SD leads to the shift of the Fraunhofer minima, prevents the critical current from dropping to zero, and causes the global CPR to experience a gradual phase shift from 0 to π state. However, accounting for nonuniform SD alone cannot explain the complex $I_{s}\left(B,\varphi_{1}\right)$ pattern observed in Fig. 2D. In particular, this model fails to account for the prominent second harmonic component present in the experimental global CPR (Fig. 2E), suggesting an intrinsic nonsinusoidal $j_{s}\left(\varphi_{1}\right)$ relation that we incorporate in our subsequent analysis.

Although the exact $j_{s}\left(\varphi_{1}\right)$ relation for our JJ cannot be measured directly, since our SQUID is not in the asymmetric regime at zero B , we assume $j_{s}\left(\varphi \right)=I_{1}\text{sin}\left(\varphi \right)+I_{2}\text{sin}\left(2\varphi \right)$ , where $I_{1}$ and $I_{2}$ are the amplitudes of the first and second harmonics, respectively. To illustrate how it affects the evolution of the global CPR, in Fig. 4 (C and D), we show the global CPR and $I_{c}\left(B\right)$ results for $j_{s}\left(\varphi \right)=1.3\text{sin}\left(\varphi \right)-0.3\text{sin}\left(2\varphi \right)$ . The resulting model accurately reproduces all experimentally observed features, namely, (i) nonreciprocal CPR (marked in Fig. 4D), (ii) JDE (Fig. 4C, top), (iii) gradual (nonsharp) 0-π transition, and (iv) peak-dip CPR structure close to the single flux quantum (yellow shaded area). The excellent agreement between our model and the experimental data confirms that the observed effects arise from the interplay between spatial distribution of the supercurrent, combined with nonsinusoidal $j_{s}\left(\varphi_{1}\right)$ dependence.

## DISCUSSION

Our theoretical analysis demonstrates that the observed anomalous CPR in our devices, its nonreciprocity, and JJ diode effect can be explained by the interplay of nonuniform current distribution and higher-harmonic contributions. A key prerequisite for the explanation is a distinct shape of $J_{c}\left(y\right)$ shown in the inset of Fig. 5. The shape is characterized by (i) its linear gradient and (ii) increased $J_{c}$ at the ends of the JJ. Although the exact origin of the observed $J_{c}\left(y\right)$ distribution remains unknown, we attribute these features to the following mechanisms. First, the slope can stem from a nonuniform gap between two superconducting NbSe_2_ parts. Taking into account a steep dependence of the critical current on the distance between the superconductors, even a small asymmetry in the junction geometry can lead to the gradient in $J_{c}\left(y\right)$ . Another possible mechanism involves Abrikosov vortices penetrating into the superconducting leads and creating nonuniform stray fields. However, trapped Abrikosov vortices would break time-reversal symmetry, violating the condition $I_{c}\left(B\right)=-I_{c}\left(-B\right)$ that is satisfied in our experiments excluding this interpretation. Second, the increased $J_{c}$ at the ends of the junction is likely related to current crowding. In our devices, the width of the superconducting NbSe_2_ leads exceeds the width of the Bi_2_Se_3_ flake, which can lead to the supercurrent streaming effect, thereby squeezing its density toward the edges (*16*, *67*). An alternative explanation involves long superconducting leads, which result in a nonlinear φ(y) dependence (*66*). In this case, the Fourier analysis of the interference pattern can produce spurious effects that mimic an apparent enhancement of supercurrent flow near the edges. Both scenarios capture the main experimental features of our data: a nonreciprocal CPR, an anomalous peak-dip structure, and the superconducting diode effect.

Next, the nonsinusoidal $j_{s}\left(\varphi_{1}\right)$ dependence can, in principle, result from both SOC and high-transparency junction. For instance, the superposition of currents from spin-dependent Andreev bound states—arising due to SOC (*68*)—can generate higher harmonics in the $j_{s}\left(\varphi_{1}\right)$ dependence (*47*). Similarly, high-transparency interfaces between the superconductor and the weak link material can also lead to nonsinusoidal behavior (*69*). Previous studies have shown that SOC has little effect on the CPR in Bi_2_Se_3_ JJ in the absence of an applied in-plane magnetic field (*50*), which is the case in our experiment. In contrast, our analysis—based on the Galaktionov-Zaikin formalism (*70*) (Supplementary Materials)—indicates that the interfaces in our device have high transparency. Therefore, the prominent second harmonic observed in the local CPR is likely related to the high transparency of the NbSe_2_/Bi_2_Se_3_ interface, which we ascribe to its atomically smooth nature achieved through our fabrication process.

It is also instructive to place the observed JDE in a broader context of nonreciprocity in superconducting systems [see (*37*) for recent review]. Originally predicted to emerge in superconducting materials with intrinsically broken inversion and time-reversal symmetries, superconducting diodes can also be engineered artificially [e.g., (*16*, *38*–*41*)]. Among numerous approaches, recently proposed SQUID-based diodes particularly stand out in terms of ease of fabrication, large diode efficiency, and remarkable tunability (*71*, *72*). The SD in our JJ1, namely, its increase at the edges, effectively mimics that of a typical SQUID loop. Together with nonsinusoidal CPR and asymmetry between the edges, this gives rise to the JDE of a conceptually similar nature as in the proposed asymmetric higher-harmonic SQUIDs (*71*, *72*).

Finally, we compare our system with the model assumed by Potter and Fu in their original prediction of the peculiar CPR structure near the flux quantum due to MBS hybridization (*32*). The theory considered a JJ based on a TI thin film in the short ( $L<\xi_{n}$ ) and narrow ( $W<\lambda_{J}$ ) limit, where both top and bottom surfaces are proximitized by a superconductor, where $\xi_{n}$ is the superconducting coherence length in Bi_2_Se_3_. Our junction satisfies these constraints, as detailed in the Supplementary Materials. Furthermore, the thin Bi_2_Se_3_ flake ( ≈5 nm) minimizes contributions from the side surfaces, while its high n-doping enables superconductivity to propagate from the top to the bottom surface through bulk states (*58*). Thus, our experimental system closely matches the conditions required by the theoretical proposal. The observed peak-dip structure (Fig. 2E) in some sense resembles the anomalous CPR due to MBS hybridizing at the edge of the junction predicted in (*32*) and can be naively attributed to their presence. Moreover, the supercurrent in this regime is expected to be close to the maximal Josephson current carried by a single quantum channel $\Delta /\Phi_{0}\approx 75$ nA for the superconducting gap of Δ∼1 meV that is fortuitously close to our experimental value at the Fraunhofer minimum—60 nA (see Fig. 2E, black curve). However, despite apparent similarity of the anomalous peak-dip CPR in the Potter-Fu model to the experimental data, there are two key differences that rule out this interpretation. First, the theoretical peak-dip structure is predicted to emerge around $\varphi_{1}=\pi$ but not at π/2 as in the case of our experiment. Second, the width of the experimental peak-dip structure is notably larger compared to the theoretical prediction (*32*). Should the MBSs be present in the Bi_2_Se_3_-based JJs, the resolution of our technique would be sufficient to reveal their signatures.

To conclude, we have demonstrated a platform for precise CPR measurements in lateral JJs composed of NbSe_2_ and few-layer Bi_2_Se_3_, enabling the reconstruction of the CPR as a function of magnetic flux. Our findings reveal unconventional CPR characteristics and a robust, tunable JDE in the vicinity of the flux quantum, stemming from nonuniform SD and nonsinusoidal local CPR. Notably, we find no evidence of MBSs near a single flux quantum, challenging previous claims in Bi_2_Se_3_-based JJs. These results establish CPR measurements as a powerful tool for probing nonreciprocal transport phenomena in superconducting systems and provide design principles for superconducting quantum devices. It would be natural to extend our approach of CPR measurements in planar vdW JJs using cracked, atomically flat superconductors to investigate other topologically nontrivial materials such as Sn-doped Bi_1.1_Sb_0.9_Te_2_S (*73*), WTe_2_ (*74*, *75*), or MnBi_2_Te_4_ (*76*) that are unstable under standard nanofabrication processing.

## MATERIALS AND METHODS

### Sample fabrication

Device fabrication was performed using a dry transfer technique in an argon-filled glovebox with controlled H_2_O and O_2_ levels (<0.5 parts per million). The fabrication process began with oxygen plasma cleaning of Si/SiO_2_ substrates to optimize the subsequent exfoliation of thin Bi_2_Se_3_ flakes. hBN, NbSe_2_, and Bi_2_Se_3_ were mechanically exfoliated onto the prepared Si/SiO_2_ substrates using adhesive tape. The heterostructure assembly proceeded by first picking up an hBN flake using a PDMS/PC (polydimethylsiloxane/polycarbonate) stamp, which was then used to pick up a cracked NbSe_2_ flake, followed by transferring the assembled stack onto the exfoliated Bi_2_Se_3_ flake.

Electrical contacts were fabricated using standard electron beam lithography, followed by CHF_3_/O_2_ reactive ion etching (RIE) of the hBN layer and electron beam evaporation of Ti/Au contacts (5 nm/65 nm). A magnetic flux line was subsequently created by depositing Ti (3 nm) followed by Al (97 nm). The SQUID loop geometry was then defined using CHF_3_/O_2_ RIE. After performing initial transport measurements on the complete SQUID, the device was divided into two separate JJs using CHF_3_/O_2_ RIE, enabling individual junction characterization after cooling. See the Supplementary Materials for details.

### Scanning transmission electron microscopy

A cross-sectional specimen of the nanodevice was prepared using a focused ion beam (FEI Versa 3D Dual Beam) after depositing a 30-nm-thick platinum layer on the surface as a protective coating. Cross-sectional STEM imaging was then performed ex situ using a JEOL ARM200F microscope equipped with an ASCOR aberration corrector, operating at 200 kV. Elemental mapping was conducted with an Oxford X-Max 100TLE EDS detector integrated with the microscope.

### Low-noise measurements

Electrical measurements were performed in a BlueFors dilution refrigerator at a base temperature of 8 mK, equipped with a 12-T superconducting solenoid. The solenoid was controlled using a Yokogawa GS610 source-measure unit. The devices were mounted on a QDevil QBoard sample holder using the BlueFors fast sample exchange system. The measurement setup incorporated two distinct wiring configurations to optimize different measurement requirements.

For precise I-V measurements, we used a low-current measurement line featuring a cryogenic filtering system consisting of a Basel Precision Instruments MFT25-100 Ohm microwave filter and thermalizer at the mixing chamber plate, complemented by a QDevil QFilter at the still plate. The setup was designed to effectively eliminate 50-Hz interference, allowing for fast and high-precision measurements. The devices were biased with a sinusoidal current (frequency: few hertz) generated by a QDevil qDAC-II and converted through a CS580 voltage-to-current converter. Both the sample voltage drop and applied voltage were amplified using a battery-powered SR560 amplifier and digitized synchronously with an NI-3232 system. Critical current determination was performed using custom LabView-based software (MeXpert) implementing a threshold method, with CS580 current offset compensation (see the Supplementary Materials for details).

For high-current applications, specifically the magnetic flux line control requiring currents up to several milliamperes, we implemented a separate configuration optimized to minimize heating effects. This setup utilized only a superconducting MFT25-25 mOhm filter at the mixing chamber, bypassing the QFilter. The circuit used aluminum superconducting bonds (additionally, QBoard resistors were removed), with current supplied by a QDevil qDAC-II through a 1-kilohm resistor.

## Supplementary Materials

### This PDF file includes:

Supplementary Text Figs. S1 to S11 References
