# Supplementary material for: Non-Majorana origin of anomalous current-phase relation and Josephson diode effect in Bi2Se3/NbSe2 Josephson junctions
Source: Sci Adv. 2025 Jun 13;11(24):eadw6925. doi: 10.1126/sciadv.adw6925 (PMC12164977; doi:10.1126/sciadv.adw6925)
Supplement: Supplementary file 1 — Supplementary Text Figs. S1 to S11 References [file sciadv.adw6925_sm.pdf]

Science Advances
AAAS

Supplementary Materials for

Non-Majorana origin of anomalous current-phase relation and Josephson diode effect in $Bi_2Se_3/NbSe_2$ Josephson junctions

Andrei Kudriashov *et al.*

Corresponding author: Andrei Kudriashov, andrei.kudriashov.97@gmail.com; Xin Zhou, xin_zhou@nus.edu.sg; Denis A. Bandurin, dab@nus.edu.sg

Sci. Adv. **11**, eadw6925 (2025)
DOI: 10.1126/sciadv.adw6925

This PDF file includes:

Supplementary Text
Figs. S1 to S11
References

Supplementary Text

Device fabrication

In this section we describe in detail the fabrication of the device shown in the main text. Other devices were fabricated using similar technological processes.

$NbSe_2/Bi_2Se_3$/hBN heterostructure

1) hBN was exfoliated at ambient conditions, using Nitto blue tape and Si/SiO_2 substrate. Flake with appropriate size and thickness was determined using an optical microscope, as shown in Fig.S1a.

2) $NbSe_2$ was exfoliated inside the Ar-field glovebox using Nitto blue tape and Si/SiO_2 substrate, heated up to 60-70°C. We were looking for flakes with a crack in it, as shown in Fig.S1b.

3) Si/SiO_2 substrates for Bi_2Se_3 were cleaned in the oxygen plasma for 15 min (50 sccm of O_2, 250 W, p=1.4e-2 mbar). Bi_2Se_3 flakes were exfoliated on the Nitto blue tape inside the glovebox and covered by another blue tape. Then, these flakes on the covered tape were taken out of the glove box and the tape was placed in contact with freshly cleaned and hot substrate at ambient conditions. Then covered by the blue tape substrate with flakes on it was put inside the glovebox and delaminated in the inert atmosphere. This process allows us to get thin Bi_2Se_3 flakes with freshly cleaved inside the glovebox surface. It should be noted, that flakes were in contact with air for a few seconds before the tape was put in contact with a freshly cleaned substrate. It may lead to slight oxidation of the bottom surface of the flakes. After this, relatively thin flakes were identified under the optical microscope. We were looking for two thin flakes with different width close to each other to use them for fabrication of two Josephson junctions in the SQUID geometry. The appropriate flakes are shown in Fig.S1c.

4) hBN was picked up using PC/PDMS stamp at 90°C. Then hBN/PC/PDMS stack was used to pick up $NbSe_2$ at 110°C. In order to not deform the crack a lot, the front was perpendicular to the crack. Then $NbSe_2$/BN/PC/PDMS was placed on top of the Bi_2Se_3 flakes so that the crack was perpendicular to the flake's edges and covered two flakes at the same time. PC was melted at 180°C and washed away with DCM, followed by IPA. The final stack is shown in Fig.S1d.

SQUID

To fabricate the final device we used a combination of standard techniques, involving electron-beam

lithography (EBL), reactive ion etching (RIE), and e-beam evaporation of the metals. The workflow of the device fabrication is shown in Fig.S2.

1) PMMA 495K A5 resist was spin-coated to the substrate and baked at 180°C for 2 min on a hotplate. Then another layer of PMMA 950K A5 was spin-coated and baked at 180°C for another 2 min on a hotplate, forming a bilayer for creating under-cut.

2) EBL was performed to make markers for the precise positioning of the design with respect to the stack.

3) Deposition mask for the flux-line was created using EBL.

4) Mild 15 sec plasma (50 sccm of O_2, 50 W, p=1.4e-2 mbar) was used to remove the resist residue. 5 nm of Ti followed by 95 nm of Al were deposited using an e-beam evaporator with rates of 0.2 Å/s and 0.1 Å/s, respectively. After the lift-off in Acetone, the flux-line was fabricated.

6) Another PMMA bilayer was spin-coated similarly and EBL was used to create a mask for contacts.

7) hBN and $NbSe_2$ were etched for 30 sec using CHF_3/O_2 RIE with flows 20 sccm and 10 sccm, respectively, and RF power of 200 W. Temperature of the stage was kept at 10°C.

8) 5 nm of Ti followed by 95 nm of Au were deposited using an e-beam evaporator with rates of 0.2 Å/s and 0.3 Å/s, respectively. After the lift-off in Acetone, the edge contacts to $NbSe_2$ were fabricated.

9) Another PMMA bilayer was spin-coated similarly and EBL was used to create a mask for the top-gates.

10) Mild 15 sec plasma (50 sccm of O_2, 50 W, p=1.4e-2 mbar) was used to remove the resist residue. 5 nm of Ti followed by 95 nm of Au were deposited using an e-beam evaporator with rates of 0.2 Å/s and 0.3 Å/s, respectively. After the lift-off in Acetone, the top gates were fabricated.

11) Another PMMA bilayer was spin-coated similarly and EBL was used to create an etching mask for the SQUID patterning.

12) hBN and $NbSe_2$ were etched for 30 sec using CHF_3/O_2 RIE with flows 20 sccm and 10 sccm, respectively, and RF power of 200 W. Temperature of the stage was kept at 10°C.

13) After the resist removal in acetone, the device fabrication is finished.

From SQUID to two individual JJs

After the transport measurements of the SQUID3 (device from the main text), we cut it into two

individual Josephson junctions in order to characterize them individually.

1) PMMA bilayer was spin-coated similarly and EBL was used to create an etching mask for the cuts.

2) The same etching recipe, as described above, was used to cut SQUID into two individual junctions, as shown in Fig.S3.

Further examples of the planal vdW Josephson junctions

The first device which was fabricated using the process described in this work is shown in Fig. S4a. It is a single Josephson junction, and its interference pattern is presented in Fig. S4b. It displays a Fraunhofer-like dependence, similar to the devices JJ1 and JJ2 shown in the main text. One notable difference is that JJ0 exhibits step-like features in its $I_c(B)$ dependence, along with hysteresis when the direction of the magnetic field sweep is reversed. We attribute this behavior to the presence of Abrikosov vortices, which complicates the CPR measurements. To solve this problem, we have observed that the etching of the $NbSe_2$ helps to prevent Abricosov vortices from influencing the measurements. It can be attributed to the creation of a potential barrier for vortex penetration or by the reduction of the total area of the superconductor, which increases the critical field at which vortices penetrate inside the superconductor (*77*).

After successful demonstration of the proximity effect, we have fabricated SQUID, which is shown in Fig. S4c. The oscillations of I_c as a function of magnetic field B for this device are shown in Fig. S4d. It demonstrates similar behavior as the device shown in the main text, namely, SQUID oscillations of I_c demonstrate pronounced second harmonics and highly non-sinusoidal oscillations. As this device was not equipped with a local magnetic flux line, an accurate measurement of the CPR was not feasible.

After that, we have fabricated two more SQUIDs with local flux line: SQUID2 and SQUID3. SQUID3 is the device from the main text, while SQUID2 was used to perform detailed STEM analysis, demonstrated in Extended figure in the main text and below.

STEM and EDS

The STEM image of two JJs are shown in Fig. S5a. As one can see, the distance between two $NbSe_2$ flakes (a crack size) is about 150 nm, and the height of hBN is about 40 nm. There is some

5 nm thick contrast region where Bi_2Se_3 is supposed to be, however, we could not get an atomic resolution. Most likely, it happened because the cut was done in a slightly wrong place: right on the edge of Bi_2Se_3. The thickness of $NbSe_2$ flake is about 12 nm, as shown in Fig.S5b.

The detailed STEM analysis of the sample SQUID2 is shown in the Fig. 1 of the main text. Elemental distribution in the junction near the edge of the $NbSe_2$ crack is shown in Fig. S5c-h.

Non-sinusoidal $j_s(\varphi)$ relation and estimation of $\xi_\mathbf{n}$

Since the SQUID device discussed in the main text is not highly asymmetric at zero magnetic field, the direct measurement of the $j_s(\varphi)$ relation is not feasible. To experimentally test for deviations of the $j_s(\varphi)$ from a purely sinusoidal form, we analyzed the temperature dependence of the critical current using the formalism developed by Galaktionov and Zaikin (*70*), by fitting it to the following equation:

$$I_s = N\frac{2\pi}{\hbar}ek_BT\sin\chi\sum_{\omega_n>0}\int_0^1 \mu\,\mathrm{d}\mu\,\frac{t_1(\mu)t_2(\mu)}{Q^{1/2}(\chi,\mu)}. \tag{S1}$$

An accurate analysis requires estimating two key characteristics of the weak link: the mean free path l and the coherence length ξ_n. At mK temperatures, the mean free path can be assumed to be larger than the length of the junction, $l > L$ (*78*). The coherence length of the Josephson junction can be estimated using the following equation for the clean limit:

$$\xi_n = \frac{\hbar v_f}{2\pi k_B T}, \tag{S2}$$

At $T_c = 3.55$ K this formula gives $\xi_n \approx 100$ nm. Thus, the junction investigated in this work can be considered in the finite length clean limit. Note that the junction is in short regime (with $\xi_n \simeq 12\ \mu$m), according to eq. S2 when T = 10 mK.

We performed the fitting of $I_c(T)$ with the following parameters: Fermi velocity $v_f = 2\cdot 10^5$ m/s for Bi_2Se_3, the number of channels $N = 91$, and the interface barrier transparency $D = 0.96$. The fitting procedure employed the Levenberg-Marquardt algorithm, incorporating experimental parameters: junction thickness $d = 200$ nm, critical temperature $T_c = 3.55$ K, and the energy gap

$\Delta = 1.76 k_B T_c$ from BCS theory.

The result of the fit is plotted in Fig. S6, together with the experimental data. The inset shows the calculated CPR at $T = 10$ mK, which deviates from $I_s(\varphi) = sin(\varphi)$, being highly skewed. It justifies two-harmonics CPR (with the second harmonic being negative), which we used in the main text to model the anomalous CPR around Fraunhofer minima, which we observed experimentally.

Interference pattern for varying W/λ_J

To test whether our Josephson junction is in the wide ($\lambda_J < W$, λ_J - Josephson penetration length) or narrow regime ($\lambda_J > W$), we compare experimentally measured $I_c(B)$ with that calculated for junctions of varying width W. To this end, we use sine-Gordon equation (*24*):

$$\frac{\partial^2 \varphi}{\partial \bar{y}^2} = \sin(\varphi) - j_b, \tag{S3}$$

with normalized $\bar{y} = y/\lambda_J$. Here j_b represents the bias current, while φ denotes the phase difference at the coordinate y. Assuming standard boundary conditions

$$\left.\frac{d\varphi}{d\bar{y}}\right|_{\bar{y}=\pm W/2} = \frac{2\pi}{W}\frac{\Phi_1}{\Phi_0}, \tag{S4}$$

we obtain $I_c(B)$ for different ratios of W/λ_J presented in Fig. S7. Here Φ_1 is the magnetic flux through JJ1 and Φ_0 is the flux quantum. The two extreme cases $W/\lambda_J = 0$ and $W/\lambda_J = 5$ can be distinguished by the notably different functional forms of $I_c(B)$ in the vicinity of zero B. In particular, at small $B < 0.75$ mT and when $W = 0$, the $I_c(B)$ dependence reproduces familiar Fraunhofer pattern $I_c(\Phi) = I_c(0)\left|\frac{\sin(\pi\Phi/\Phi_0)}{\pi\Phi/\Phi_0}\right|$. On the contrary, the zero-order Fraunhofer peak is notably wider for the of $W/\lambda_J = 5$ so that even the first Fraunhofer minimum is obscured. When compared to the experiment, the width of the measured $I_c(B)$ peak around $B = 0$ follows closely the $W = 0$ model indicating that our JJ1 being in the narrow regime. As explained in the main text, the overall deviation of the $I_c(B)$ from the standard Fraunhofer pattern originates from the non-uniform supercurrent distribution.

Non-linear $\varphi(y)$ and geometric effects

The functional form of $I_c(B)$ is governed by the geometry of the superconducting electrodes, particularly the length-to-width ratio L/W (*66*). This is because the phase profile $\varphi(y)$ is approximately

linear only when $L/W \ll 1$, but becomes increasingly nonlinear as L/W grows. Following the formalism introduced in Ref. (*66*), we calculated $I_c(B)$ for a range of L/W values, assuming a uniform supercurrent distribution, and reconstructed $J_c(y)$ using the same Fourier algorithm employed in the main text, as shown in Fig. S9. As the figure demonstrates, the reconstruction introduces an artifact in the form of artificially enhanced $J_c(y)$ near the junction edges when $L/W > 0.5$. In this regime, the Fourier transform method can no longer reliably capture the true supercurrent distribution.

We further note that the main effects observed in this work (the peak-dip structure in the CPR, non-reciprocal CPR, and diode effects) can thus, in principle, be explained without invoking an increased supercurrent density at the edges. Instead, they may originate from a non-linear dependence of φ on the coordinate y along the junction, as demonstrated in Fig. S10.

Current source offset compensation

For accurate measurements of the superconducting diode effect, it is crucial to know the exact value of the applied current. However, a DC offset is inevitably present in all current sources due to technical limitations. To measure and compensate for the current offset of the voltage-controlled current source CS580, the following procedure was implemented. A 1 MOhm resistor was connected to the output of the voltage-controlled current source CS580, with both input and output disabled. The voltage drop across the resistor was amplified using the SR560 voltage amplifier, and the amplified voltage was measured using the NI-3232, as shown in the inset of Fig. S11. Since the voltage amplifiers also exhibit a DC offset, this offset was subtracted from subsequent readings (because in this configuration, no current can flow, and therefore, the actual voltage drop across the resistor should be zero).

Fig. S11 shows the measured current flowing through 1 MOhm resistor. As one can see, when the output of CS580 is disabled, no current is flowing. However, when the output was enabled at approximately 40 seconds after the beginning of the experiment, a finite current of about 2 nA started to flow. Current increases to 32 nA when the input (with QDevil qDAC-II connected to it) is enabled at approximately 65 sec.

To compensate for this finite current, -32 nA DC current was added to the output using the offset compensation function of the CS580.

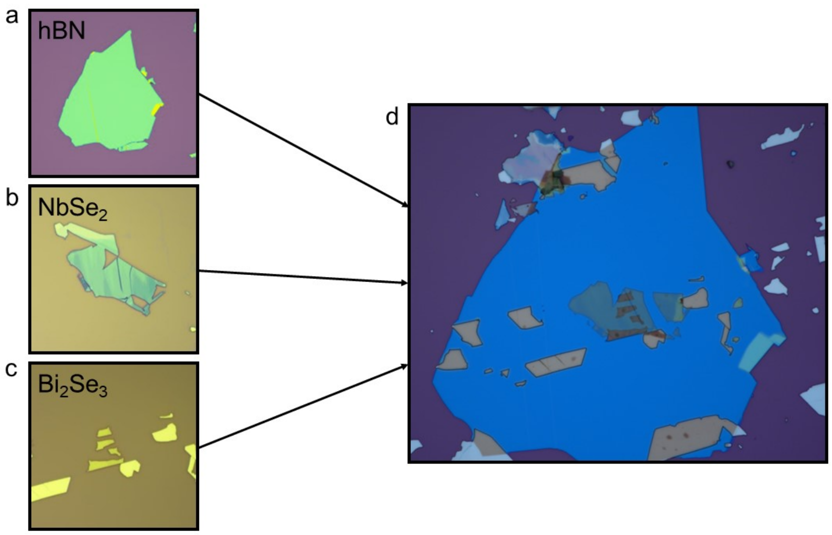

Figure S1: **Fabrication of the stack for the main device. a,** An optical micrograph of the hBN flake. **b,** An optical micrograph of the $NbSe_2$ flake. **c,** An optical micrograph of the Bi_2Se_3 flake. **d,** An optical micrograph of the final stack after the removal of melted PC.

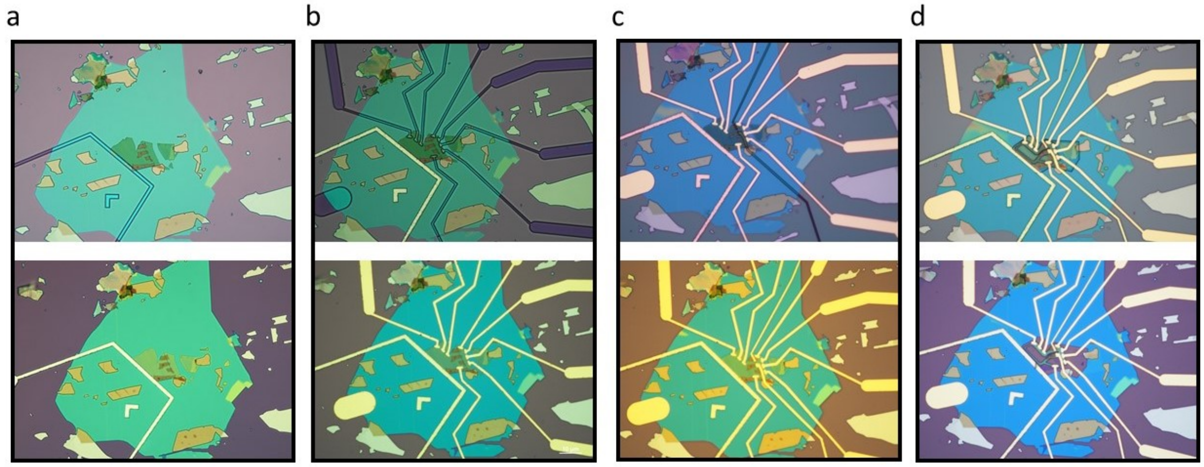

Figure S2: **Fabrication of the main device. a,** EBL (top panel) and deposition (lower panel) of the local flax line. **b,** EBL (top panel) and deposition (lower panel) of the contacts. **c,** EBL (top panel) and deposition (lower panel) of the top gates. **d,** EBL (top panel) and etching (lower panel) of the SQUID geimetry.

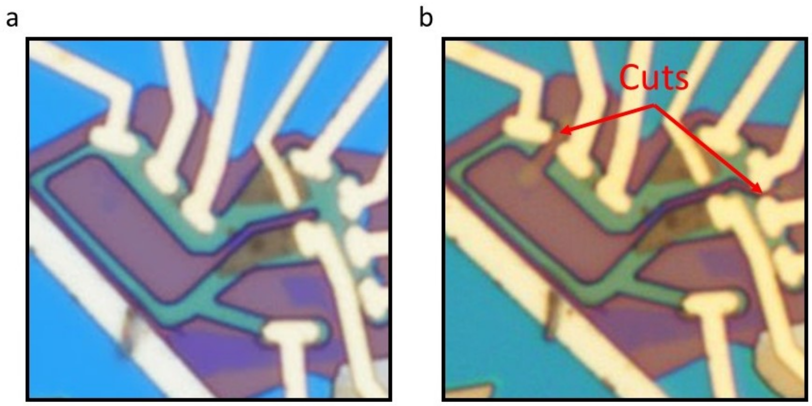

Figure S3: **From SQUID3 to JJ1 and JJ2. a,** Optical photograph of the SQUID3 device discussed in the main text. **b,** Two individual Josephson junctions JJ1 and JJ2 obtained after etching the SQUID into two not-connected devices.

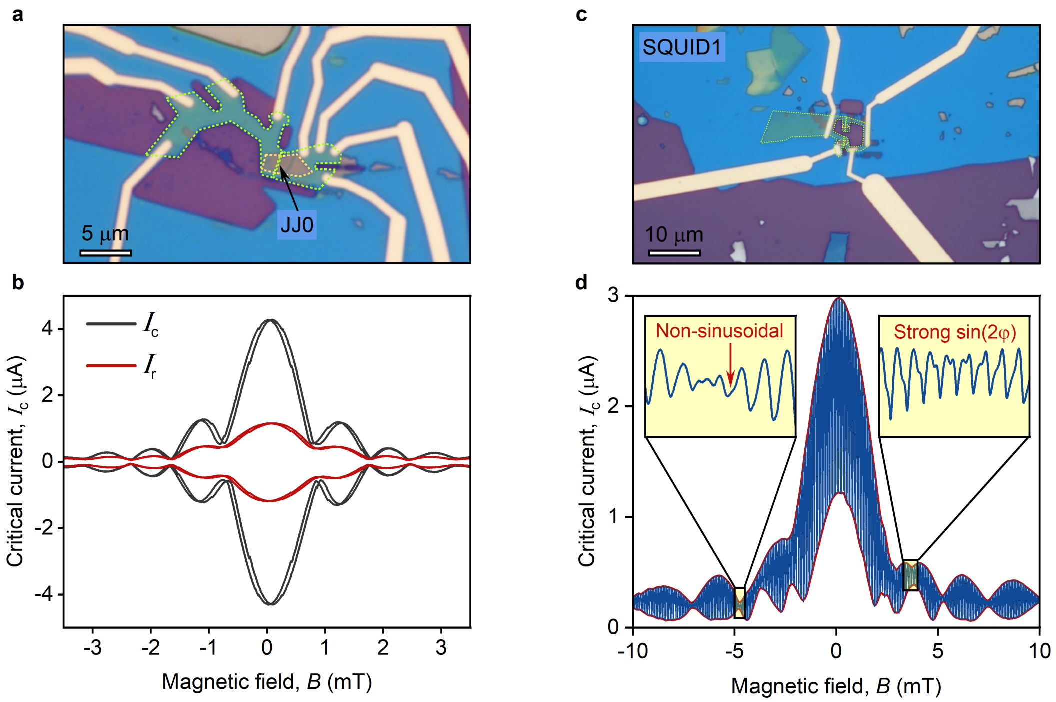

Figure S4: **Other devices. a,** Optical micrograph of the sample named JJ0. Green and yellow dashed lines highlight $NbSe_2$ and Bi_2Se_3 flakes, respectively. **b,** Critical current I_c and retrapping current I_r of device JJ0 plotted versus magnetic field B. **c,** Optical micrograph of the sample named SQUID1. Green and yellow dashed lines highlight $NbSe_2$ and Bi_2Se_3 flakes, respectively. **d,** Critical current I_c of device SQUID1 (blue line) plotted versus magnetic field B. Red lines show the envelope functions. The inserts display zoomed-in views of the SQUID oscillations near the minima of the interference pattern.

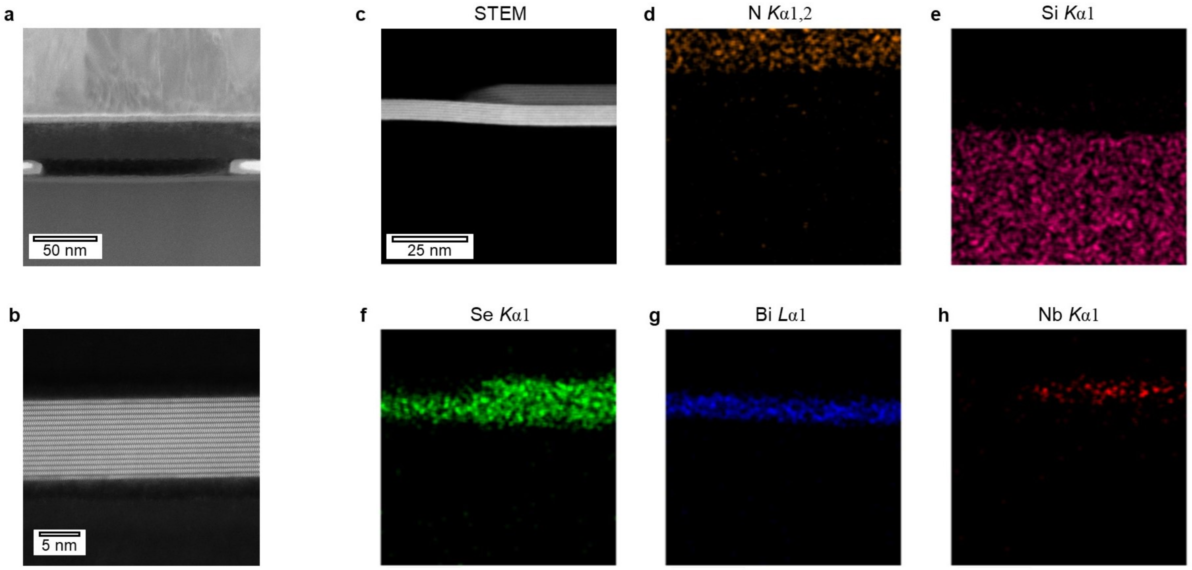

Figure S5: **STEM and EDS analysis a,** STEM of the junction region of JJ2. **b,** HAADF-STEM of the $NbSe_2$ region. **c,** STEM image of the junction region of SQUID2 near the edge of the $NbSe_2$ crack. **d-h,** Energy dispersive spectroscopy (EDS) mapping of the same region showing distributions of Nitrogen, Silicon, Selenium, Bismuth, and Niobium, respectively.

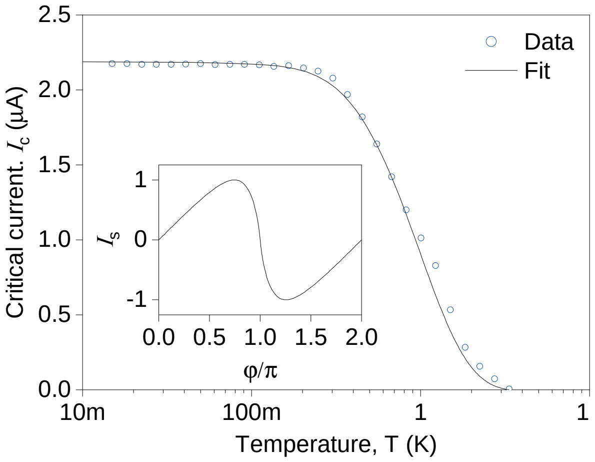

Figure S6: **Results of the $I_c(T)$ modeling** Critical current of JJ1 as a function of temperature. Blue dots show the experimental data, a black line is a fit by Galaktionov and Zaikin model. Inset shows theoretically determined CPR.

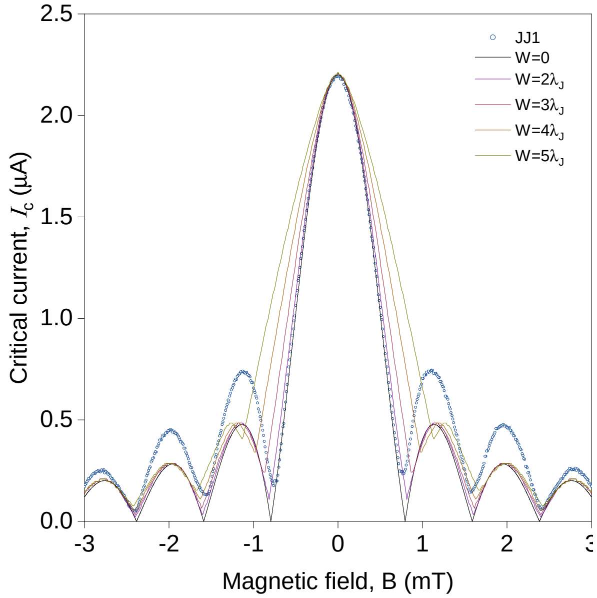

Figure S7: **Interference pattern for varying** W/λ_J. Symbols: Experimental $I_c(B)$ for JJ1. Solid lines: Calculated $I_c(B)$ dependecies for given W/λ_J.

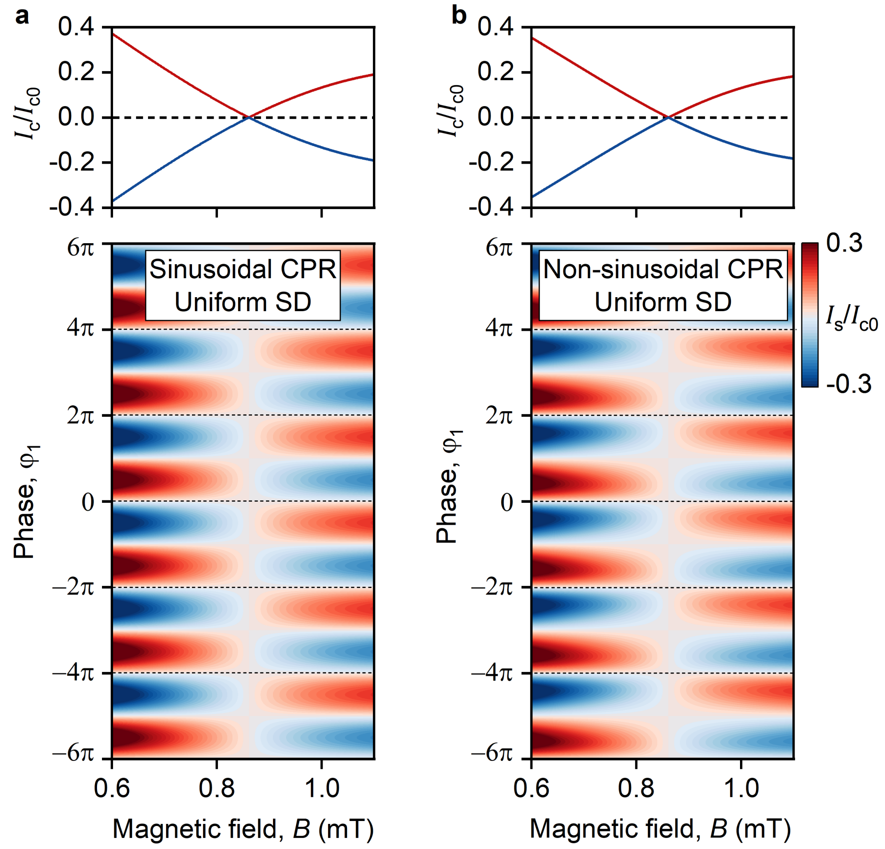

Figure S8: **Results of the $I_s(B, \varphi_1)$ modeling. (A-B)** Calculated positive I_c^+ and negative I_c^- critical currents of the JJ as a function of magnetic field B (upper panel) and calculated supercurrent I_s as a function of phase difference φ_1 and magnetic field B (lower panel). **(A)** The case of sinusoidal current-phase relation and uniform supercurrent distribution. **(B)** The case of non-sinusoidal current-phase relation and uniform supercurrent distribution.

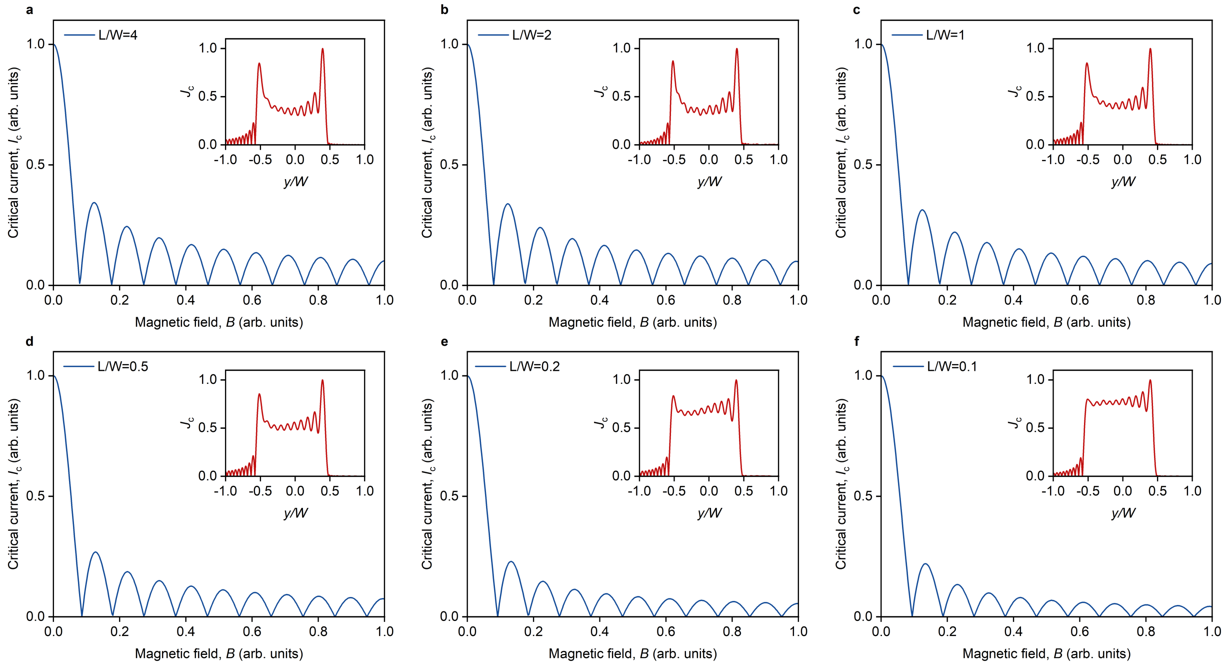

Figure S9: **artifact of $J_c(y)$ reconstruction in the case of planar geometry.** Calculated $I_c(B)$ (blue line) for different L/W ratios and corresponding reconstracted $J_c(y)$ (red line).

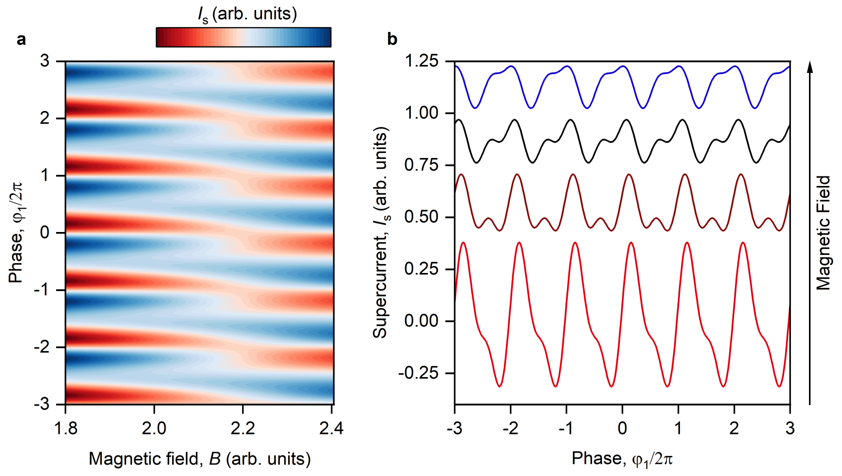

Figure S10: **Results of the calculations of $I_s(\varphi_1, B)$ assuming non-linear $\varphi(y)$. (A)** Calculated supercurrent I_s as a function of phase difference φ_1 and magnetic field B for $L/W = 1$, non-sinusoidal CPR $I_s(\varphi) = 1.3\sin(\varphi) - 0.3\sin(\varphi)$ and assymetric supercurent distribution $J_c = 1 + 0.2(y - W/2)/W$. **(B)** Global CPR $I_s(\varphi_1)$ for selected values of magnetic field.

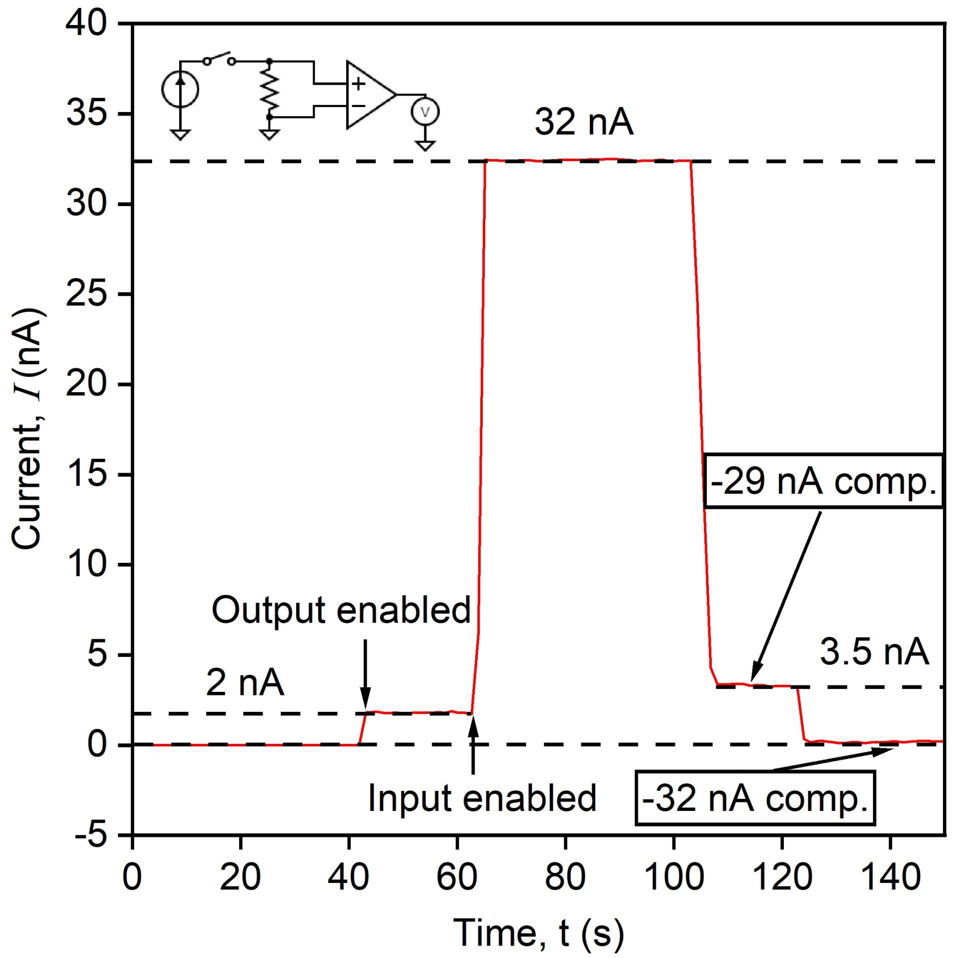

Figure S11: **Offset compensation.** Measured value of output current as a function of time. Inset shows the measurement circuit.

REFERENCES AND NOTES

1. M. H. Devoret, R. J. Schoelkopf, Superconducting circuits for quantum information: An outlook. *Science* **339**, 1169–1174 (2013).

2. H. B. Wang, S. Guénon, B. Gross, J. Yuan, Z. G. Jiang, Y. Y. Zhong, M. Grünzweig, A. Iishi, P. H. Wu, T. Hatano, D. Koelle, R. Kleiner, Coherent terahertz emission of intrinsic Josephson junction stacks in the hot spot regime. *Phys. Rev. Lett.* **105**, 057002 (2010).

3. D. S. Holmes, A. L. Ripple, M. A. Manheimer, Energy-efficient superconducting computing–power budgets and requirements. *IEEE Trans. Appl. Supercond.* **23**, 1701610 (2013).

4. G.-H. Lee, D. K. Efetov, W. Jung, L. Ranzani, E. D. Walsh, T. A. Ohki, T. Taniguchi, K. Watanabe, P. Kim, D. Englund, K. C. Fong, Graphene-based Josephson junction microwave bolometer. *Nature* **586**, 42–46 (2020).

5. A. Y. Kitaev, Unpaired Majorana fermions in quantum wires. *Phys. Uspekhi* **44**, 131–136 (2001).

6. R. M. Lutchyn, J. D. Sau, S. Das Sarma, Majorana fermions and a topological phase transition in semiconductor-superconductor heterostructures. *Phys. Rev. Lett.* **105**, 077001 (2010).

7. P. A. Ioselevich, P. M. Ostrovsky, M. V. Feigel'man, Majorana state on the surface of a disordered three-dimensional topological insulator. *Phys. Rev. B* **86**, 035441 (2012).

8. J. Alicea, New directions in the pursuit of Majorana fermions in solid state systems. *Rep. Prog. Phys.* **75**, 076501 (2012).

9. C. W. J. Beenakker, Search for majorana fermions in superconductors. *Annu. Rev. Condens. Matter Phys.* **4**, 113–136 (2013).

10. S. Kezilebieke, M. N. Huda, V. Vaňo, M. Aapro, S. C. Ganguli, O. J. Silveira, S. Głodzik, A. S. Foster, T. Ojanen, P. Liljeroth, Topological superconductivity in a van der Waals heterostructure. *Nature* **588**, 424–428 (2020).

11. P. San-Jose, J. L. Lado, R. Aguado, F. Guinea, J. Fernández-Rossier, Majorana zero modes in graphene. *Phys. Rev. X* **5**, 041042 (2015).

12. M. Alidoust, Critical supercurrent and φ0 state for probing a persistent spin helix. *Phys. Rev. B* **101**, 155123 (2020).

13. A. Diez-Carlon, J. Diez-Merida, P. Rout, D. Sedov, P. Virtanen, S. Banerjee, R. P. S. Penttila, P. Altpeter, K. Watanabe, T. Taniguchi, S.-Y. Yang, K. T. Law, T. T. Heikkila, P. Torma, M. S. Scheurer, D. K. Efetov, Probing the flat-band limit of the superconducting proximity effect in twisted bilayer graphene Josephson junctions. arXiv:2502.04785 [cond-mat.supr-con] (2025).

14. Q. Li, Y. N. Tsay, M. Suenaga, R. A. Klemm, G. D. Gu, N. Koshizuka, $Bi_2Sr_2CaCuO_{8+\delta}$ bicrystal *c*-axis twist Josephson junctions: A new phase-sensitive test of order parameter symmetry. *Phys. Rev. Lett.* **83**, 4160–4163 (1999).

15. D. Roditchev, C. Brun, L. Serrier-Garcia, J. C. Cuevas, V. H. L. Bessa, M. V. Milošević, F. Debontridder, V. Stolyarov, T. Cren, Direct observation of Josephson vortex cores. *Nat. Phys.* **11**, 332–337 (2015).

16. S. Chen, S. Park, U. Vool, N. Maksimovic, D. A. Broadway, M. Flaks, T. X. Zhou, P. Maletinsky, A. Stern, B. I. Halperin, A. Yacoby, Current induced hidden states in Josephson junctions. *Nat. Commun.* **15**, 8059 (2024).

17. A. Gutfreund, H. Matsuki, V. Plastovets, A. Noah, L. Gorzawski, N. Fridman, G. Yang, A. Buzdin, O. Millo, J. W. A. Robinson, Y. Anahory, Direct observation of a superconducting vortex diode. *Nat. Commun.* **14**, 1630 (2023).

18. J. Williams, A. J. Bestwick, P. Gallagher, S. S. Hong, Y. Cui, A. S. Bleich, J. G. Analytis, I. R. Fisher, D. Goldhaber-Gordon, Unconventional Josephson effect in hybrid superconductor-topological insulator devices. *Phys. Rev. Lett.* **109**, 056803 (2012).

19. M. Veldhorst, M. Snelder, M. Hoek, T. Gang, V. K. Guduru, X. L. Wang, U. Zeitler, W. G. van der Wiel, A. A. Golubov, H. Hilgenkamp, A. Brinkman, Josephson supercurrent through a topological insulator surface state. *Nat. Mater.* **11**, 417–421 (2012).

20. S. Ghatak, O. Breunig, F. Yang, Z. Wang, A. A. Taskin, Y. Ando, Anomalous Fraunhofer patterns in gated Josephson junctions based on the bulk-insulating topological insulator $BiSbTeSe_2$. *Nano Lett.* **18**, 5124–5131 (2018).

21. P. A. Ioselevich, M. V. Feigel'man, Anomalous Josephson current via majorana bound states in topological insulators. *Phys. Rev. Lett.* **106**, 077003 (2011).

22. R. C. Dynes, T. A. Fulton, Supercurrent density distribution in Josephson junctions. *Phys. Rev. B* **3**, 3015–3023 (1971).

23. C. Owen, D. Scalapino, Vortex structure and critical currents in Josephson junctions. *Phys. Rev.* **164**, 538–544 (1967).

24. B. Antonio, P. Gianfranco, *Physics and Applications of the Josephson Effect* (John Wiley & Sons Inc., 2006).

25. R. A. Hovhannisyan, T. Golod, V. M. Krasnov, Controllable manipulation of semifluxon states in phase-shifted Josephson junctions. *Phys. Rev. Lett.* **132**, 227001 (2024).

26. T. Golod, V. M. Krasnov, Demonstration of a superconducting diode-with-memory, operational at zero magnetic field with switchable nonreciprocity. *Nat. Commun.* **13**, 3658 (2022).

27. T. Golod, A. Pagliero, V. M. Krasnov, Two mechanisms of Josephson phase shift generation by an Abrikosov vortex. *Phys. Rev. B* **100**, 174511 (2019).

28. M. Foltyn, K. Norowski, A. Savin, M. Zgirski, Quantum thermodynamics with a single superconducting vortex. *Sci. Adv.* **10**, eado4032 (2024).

29. A. Rashidi, W. Huynh, B. Guo, S. Ahadi, S. Stemmer, Vortex-induced anomalies in the superconducting quantum interference patterns of topological insulator Josephson junctions. *NPJ Quantum Mater.* **9**, 70 (2024).

30. R. A. Hovhannisyan, T. Golod, V. M. Krasnov, Superresolution magnetic imaging by a Josephson junction via holographic reconstruction of $I_c(H)$ modulation. *Phys. Rev. Appl.* **20**, 064012 (2023).

31. L. Fu, C. L. Kane, Superconducting proximity effect and majorana fermions at the surface of a topological insulator. *Phys. Rev. Lett.* **100**, 096407 (2008).

32. A. C. Potter, L. Fu, Anomalous supercurrent from Majorana states in topological insulator Josephson junctions. *Phys. Rev. B* **88**, 121109 (2013).

33. S. S. Hegde, G. Yue, Y. Wang, E. Huemiller, D. J. Van Harlingen, S. Vishveshwara, A topological Josephson junction platform for creating, manipulating, and braiding Majorana bound states. *Ann. Phys. Rehabil. Med.* **423**, 168326 (2020).

34. K. Laubscher, J. D. Sau, Detection of Majorana zero modes bound to Josephson vortices in planar S-TI-S junctions. arXiv:2411.00756 [cond-mat.mes-hall] (2024).

35. J. D. Sau, R. M. Lutchyn, S. Tewari, S. Das Sarma, Generic new platform for topological quantum computation using semiconductor heterostructures. *Phys. Rev. Lett.* **104**, 040502 (2010).

36. C. Nayak, S. H. Simon, A. Stern, M. Freedman, S. Das Sarma, Non-Abelian anyons and topological quantum computation. *Rev. Mod. Phys.* **80**, 1083–1159 (2008).

37. M. Nadeem, M. S. Fuhrer, X. Wang, The superconducting diode effect. *Nat. Rev. Phys.* **5**, 558–577 (2023).

38. H. Wu, Y. Wang, Y. Xu, P. K. Sivakumar, C. Pasco, U. Filippozzi, S. S. P. Parkin, Y.-J. Zeng, T. McQueen, M. N. Ali, The field-free Josephson diode in a van der Waals heterostructure. *Nature* **604**, 653–656 (2022).

39. Y.-Y. Lyu, J. Jiang, Y.-L. Wang, Z.-L. Xiao, S. Dong, Q.-H. Chen, M. V. Milošević, H. Wang, R. Divan, J. E. Pearson, P. Wu, F. M. Peeters, W.-K. Kwok, Superconducting diode effect via conformal-mapped nanoholes. *Nat. Commun.* **12**, 2703 (2021).

40. F. Ando, Y. Miyasaka, T. Li, J. Ishizuka, T. Arakawa, Y. Shiota, T. Moriyama, Y. Yanase, T. Ono, Observation of superconducting diode effect. *Nature* **584**, 373–376 (2020).

41. S. Reinhardt, T. Ascherl, A. Costa, J. Berger, S. Gronin, G. C. Gardner, T. Lindemann, M. J. Manfra, J. Fabian, D. Kochan, C. Strunk, N. Paradiso, Link between supercurrent diode and anomalous Josephson effect revealed by gate-controlled interferometry. *Nat. Commun.* **15**, 4413 (2024).

42. C.-Z. Chen, J. J. He, M. N. Ali, G. H. Lee, K. C. Fong, K. T. Law, Asymmetric Josephson effect in inversion symmetry breaking topological materials. *Phys. Rev. B* **98**, 075430 (2018).

43. J. Cayao, N. Nagaosa, Y. Tanaka, Enhancing the Josephson diode effect with Majorana bound states. *Phys. Rev. B* **109**, L081405 (2024).

44. Z. Liu, L. Huang, J. Wang, Josephson diode effect in topological superconductors. *Phys. Rev. B* **110**, 014519 (2024).

45. Y. Xia, D. Qian, D. Hsieh, L. Wray, A. Pal, H. Lin, A. Bansil, D. Grauer, Y. S. Hor, R. J. Cava, M. Z. Hasan, Observation of a large-gap topological-insulator class with a single Dirac cone on the surface. *Nat. Phys.* **5**, 398–402 (2009).

46. X.-L. Qi, S.-C. Zhang, Topological insulators and superconductors. *Rev. Mod. Phys.* **83**, 1057–1110 (2011).

47. I. Sochnikov, L. Maier, C. A. Watson, J. R. Kirtley, C. Gould, G. Tkachov, E. M. Hankiewicz, C. Brüne, H. Buhmann, L. W. Molenkamp, K. A. Moler, Nonsinusoidal current-phase relationship in Josephson junctions from the 3D topological insulator HgTe. *Phys. Rev. Lett.* **114**, 066801 (2015).

48. I. Sochnikov, A. J. Bestwick, J. R. Williams, T. M. Lippman, I. R. Fisher, D. Goldhaber-Gordon, J. R. Kirtley, K. A. Moler, Direct measurement of current-phase relations in superconductor/topological insulator/superconductor junctions. *Nano Lett.* **13**, 3086–3092 (2013).

49. M. Kayyalha, M. Kargarian, A. Kazakov, I. Miotkowski, V. M. Galitski, V. M. Yakovenko, L. P. Rokhinson, Y. P. Chen, Anomalous low-temperature enhancement of supercurrent in topological-insulator nanoribbon Josephson junctions: Evidence for low-energy Andreev bound states. *Phys. Rev. Lett.* **122**, 047003 (2019).

50. A. Assouline, C. Feuillet-Palma, N. Bergeal, T. Zhang, A. Mottaghizadeh, A. Zimmers, E. Lhuillier, M. Eddrie, P. Atkinson, M. Aprili, H. Aubin, Spin-orbit induced phase-shift in Bi_2Se_3 Josephson junctions. *Nat. Commun.* **10**, 126 (2019).

51. I. Babich, A. Kudriashov, D. Baranov, V. S. Stolyarov, Limitations of the current–phase relation measurements by an asymmetric DC-SQUID. *Nano Lett.* **23**, 6713–6719 (2023).

52. M. Endres, A. Kononov, H. S. Arachchige, J. Yan, D. Mandrus, K. Watanabe, T. Taniguchi, C. Schönenberger, Current–phase relation of a WTe_2 Josephson junction. *Nano Lett.* **23**, 4654–4659 (2023).

53. K. Mazumder, P. M. Shirage, A brief review of Bi_2Se_3 based topological insulator: From fundamentals to applications. *J. Alloys Compd.* **888**, 161492 (2021).

54. Y. Cao, A. Mishchenko, G. L. Yu, E. Khestanova, A. P. Rooney, E. Prestat, A. V. Kretinin, P. Blake, M. B. Shalom, C. Woods, J. Chapman, G. Balakrishnan, I. V. Grigorieva, K. S. Novoselov, B. A. Piot, M. Potemski, K. Watanabe, T. Taniguchi, S. J. Haigh, A. K. Geim, R. V. Gorbachev, Quality heterostructures from two-dimensional crystals unstable in air by their assembly in inert atmosphere. *Nano Lett.* **15**, 4914–4921 (2015).

55. A. Zalic, T. Taniguchi, K. Watanabe, S. Gazit, H. Steinberg, High magnetic field stability in a planar graphene-$NbSe_2$ SQUID. *Nano Lett.* **23**, 6102–6108 (2023).

56. T. Dvir, A. Zalic, E. H. Fyhn, M. Amundsen, T. Taniguchi, K. Watanabe, J. Linder, H. Steinberg, Planar graphene-$NbSe_2$ Josephson junctions in a parallel magnetic field. *Phys. Rev. B* **103**, 115401 (2021).

57. A. Kudriashov, I. Babich, R. A. Hovhannisyan, A. G. Shishkin, S. N. Kozlov, A. Fedorov, D. V. Vyalikh, E. Khestanova, M. Y. Kupriyanov, V. S. Stolyarov, Revealing intrinsic superconductivity of the Nb/BiSbTe$_2$Se interface. *Adv. Funct. Mater.* **32**, 2209853 (2022).

58. M.-X. Wang, C. Liu, J.-P. Xu, F. Yang, L. Miao, M.-Y. Yao, C. L. Gao, C. Shen, X. Ma, X. Chen, Z.-A. Xu, Y. Liu, S.-C. Zhang, D. Qian, J.-F. Jia, Q.-K. Xue, The coexistence of superconductivity and topological order in the Bi$_2$Se$_3$ thin films. *Science* **336**, 52–55 (2012).

59. N. Yabuki, R. Moriya, M. Arai, Y. Sata, S. Morikawa, S. Masubuchi, T. Machida, Supercurrent in van der Waals Josephson junction. *Nat. Commun.* **7**, 10616 (2016).

60. S. Son, Y. J. Shin, K. Zhang, J. Shin, S. Lee, H. Idzuchi, M. J. Coak, H. Kim, J. Kim, J. H. Kim, M. Kim, D. Kim, P. Kim, J.-G. Park, Strongly adhesive dry transfer technique for van der Waals heterostructure. *2D Mater.* **7**, 041005 (2020).

61. H. Courtois, M. Meschke, J. Peltonen, J. P. Pekola, Origin of hysteresis in a proximity Josephson junction. *Phys. Rev. Lett.* **101**, 067002 (2008).

62. V. M. Krasnov, T. Golod, T. Bauch, P. Delsing, Anticorrelation between temperature and fluctuations of the switching current in moderately damped Josephson junctions. *Phys. Rev. B Condens. Matter Mater. Phys.* **76**, 224517 (2007).

63. H. Miyazaki, A. Kanda, Y. Ootuka, Current-phase relation of a superconducting quantum point contact. *Phys. C Superconduct. Appl.* **437-438**, 217–219 (2006).

64. M. L. Della Rocca, M. Chauvin, B. Huard, H. Pothier, D. Esteve, C. Urbina, Measurement of the current-phase relation of superconducting atomic contacts. *Phys. Rev. Lett.* **99**, 127005 (2007).

65. E. Goldobin, D. Koelle, R. Kleiner, A. I. Buzdin, Josephson junctions with second harmonic in the current-phase relation: Properties of φ junctions. *Phys. Rev. B* **76**, 224523 (2007).

66. J. R. Clem, Josephson junctions in thin and narrow rectangular superconducting strips. *Phys. Rev. B* **81**, 144515 (2010).

67. H. L. Hortensius, E. F. C. Driessen, T. M. Klapwijk, K. K. Berggren, J. R. Clem, Critical-current reduction in thin superconducting wires due to current crowding. *Appl. Phys. Lett.* **100**, 182602 (2012).

68. L. Tosi, C. Metzger, M. F. Goffman, C. Urbina, H. Pothier, S. Park, A. L. Yeyati, J. Nygård, P. Krogstrup, Spin-orbit splitting of Andreev states revealed by microwave spectroscopy. *Phys. Rev. X* **9**, 011010 (2019).

69. A. A. Golubov, M. Y. Kupriyanov, E. Il'Ichev, The current-phase relation in Josephson junctions. *Rev. Mod. Phys.* **76**, 411–469 (2004).

70. A. V. Galaktionov, A. D. Zaikin, Quantum interference and supercurrent in multiple-barrier proximity structures. *Phys. Rev. B* **65**, 184507 (2002).

71. R. S. Souto, M. Leijnse, C. Schrade, Josephson diode effect in supercurrent interferometers. *Phys. Rev. Lett.* **129**, 267702 (2022).

72. Y. V. Fominov, D. Mikhailov, Asymmetric higher-harmonic SQUID as a Josephson diode. *Phys. Rev. B* **106**, 134514 (2022).

73. S. K. Kushwaha, I. Pletikosić, T. Liang, A. Gyenis, S. H. Lapidus, Y. Tian, H. Zhao, K. S. Burch, J. Lin, W. Wang, H. Ji, A. V. Fedorov, A. Yazdani, N. P. Ong, T. Valla, R. J. Cava, Sn-doped $Bi_{1.1}Sb_{0.9}Te_2S$ bulk crystal topological insulator with excellent properties. *Nat. Commun.* **7**, 11456 (2016).

74. S. Wu, V. Fatemi, Q. D. Gibson, K. Watanabe, T. Taniguchi, R. J. Cava, P. Jarillo-Herrero, Observation of the quantum spin Hall effect up to 100 kelvin in a monolayer crystal. *Science* **359**, 76–79 (2018).

75. Y.-B. Choi, Y. Xie, C. Z. Chen, J. Park, S. B. Song, J. Yoon, B. J. Kim, T. Taniguchi, K. Watanabe, J. Kim, K. C. Fong, M. N. Ali, K. T. Law, G. H. Lee, Evidence of higher-order topology in multilayer WTe_2 from Josephson coupling through anisotropic hinge states. *Nat. Mater.* **19**, 974–979 (2020).

76. Y. Deng, Y. Yu, M. Z. Shi, Z. Guo, Z. Xu, J. Wang, X. H. Chen, Y. Zhang, Quantum anomalous Hall effect in intrinsic magnetic topological insulator $MnBi_2Te_4$. *Science* **367**, 895–900 (2020).

77. G. Stan, S. B. Field, J. M. Martinis, Critical field for complete vortex expulsion from narrow superconducting strips. *Phys. Rev. Lett.* **92**, 097003 (2004).

78. V. S. Kamboj, A. Singh, T. Ferrus, H. E. Beere, L. B. Duffy, T. Hesjedal, C. H. W. Barnes, D. A. Ritchie, Probing the topological surface state in Bi_2Se_3 thin films using temperature-dependent terahertz spectroscopy. *ACS Photonics* **4**, 2711–2718 (2017).
